# Supplementary material for: Mitochondrial genomic alterations in cholangiocarcinoma cell lines
Source: PLoS One. 2025 Jun 9;20(6):e0323844. doi: 10.1371/journal.pone.0323844 (PMC12148147; doi:10.1371/journal.pone.0323844)
Supplement: S1 File — (DOCX) [file pone.0323844.s001.docx]

**S1 Table. Genomic DNA concentration.** These DNA concentrations were measured by Qubit fluorometer with dsDNA HS assay.

| Cell name | Replicate | dsDNA conc. (ng/µL) |
| --- | --- | --- |
| KKU-213A | Tube 1 | 95.8 |
|  | Tube 2 | 112 |
|  | Tube 3 | 81.2 |
| KKU-100 | Tube 1 | 32.2 |
|  | Tube 2 | 37.8 |
|  | Tube 3 | 29 |
| KKU-055 | Tube 1 | 81.6 |
|  | Tube 2 | 60.4 |
|  | Tube 3 | 73.4 |
| KKU-452 | Tube 1 | 50.8 |
|  | Tube 2 | 112 |
|  | Tube 3 | 115.6 |
| KKU-023 | Tube 1 | 43.2 |
|  | Tube 2 | 132 |
|  | Tube 3 | 112 |
| MMNK-1 | Tube 1 | 118.8 |
|  | Tube 2 | 53.4 |
|  | Tube 3 | 110 |

**S2 Table. Genomic DNA concentration.** These DNA concentrations were measured by NanoDrop spectrophotometer.

| Cell name | Replicate | Nucleic acid (ng/µL) | 260/280 | 260/230 |
| --- | --- | --- | --- | --- |
| KKU-213A | Tube 1 | 406.2 | 2.04 | 2.28 |
|  | Tube 2 | 394.2 | 2.05 | 2.24 |
|  | Tube 3 | 496.7 | 2.06 | 2.22 |
| KKU-100 | Tube 1 | 31.2 | 2.18 | 1.13 |
|  | Tube 2 | 23.9 | 2.22 | 1.1 |
|  | Tube 3 | 39.2 | 2.16 | 1.25 |
| KKU-055 | Tube 1 | 108.5 | 2.02 | 2.12 |
|  | Tube 2 | 204.7 | 2.06 | 2.21 |
|  | Tube 3 | 78.7 | 1.97 | 1.79 |
| KKU-452 | Tube 1 | 135.9 | 1.93 | 1.64 |
|  | Tube 2 | 75.2 | 1.95 | 2.03 |
|  | Tube 3 | 86.2 | 1.92 | 1.58 |
| KKU-023 | Tube 1 | 134.2 | 2.12 | 2.15 |
|  | Tube 2 | 323.9 | 2.08 | 2.03 |
|  | Tube 3 | 478.8 | 2.05 | 1.93 |
| MMNK-1 | Tube 1 | 74.9 | 1.91 | 1.85 |
|  | Tube 2 | 46.2 | 1.84 | 1.15 |
|  | Tube 3 | 69.9 | 1.93 | 1.68 |

**S3 Table. Circular DNA concentration.** These circular DNA concentrations were measured by Qubit fluorometer with dsDNA HS assay.

| Cell name | Replicate | dsDNA conc. (ng/µL) | |
| --- | --- | --- | --- |
| KKU-213A | Tube 1 | 75.2 | |
|  | Tube 2 | 108 | |
|  | Tube 3 | 332.8 | |
| KKU-100 | Tube 1 | 30.4 | |
|  | Tube 2 | 37.8 | |
|  | Tube 3 | 11.1 | |
| KKU-055 | Tube 1 | 38.0 | |
|  | Tube 2 | 71.2 | |
|  | Tube 3 | 93.0 | |
| KKU-452 | Tube 1 | | 118 |
|  | Tube 2 | | 4.64 |
|  | Tube 3 | | 3.72 |
| KKU-023 | Tube 1 | | 43.2 |
|  | Tube 2 | | 62.8 |
|  | Tube 3 | | 72.8 |
| MMNK-1 | Tube 1 | | 9.30 |
|  | Tube 2 | | 20.0 |
|  | Tube 3 | | 3.38 |

**S4 Table. Post-MDA mtDNA concentration.** These mtDNA concentrations were measured by Qubit fluorometer with dsDNA HS assay.

| Cell name | Replicate | dsDNA conc. (ng/µL) | |
| --- | --- | --- | --- |
| KKU-213A | Tube 1 | 63.8 | |
|  | Tube 2 | 81.8 | |
|  | Tube 3 | 62.2 | |
| KKU-100 | Tube 1 | 159.8 | |
|  | Tube 2 | 118 | |
|  | Tube 3 | 100.4 | |
| KKU-055 | Tube 1 | 133.6 | |
|  | Tube 2 | 112 | |
|  | Tube 3 | 112 | |
| KKU-452 | Tube 1 | | 78.4 |
|  | Tube 2 | | 71.2 |
|  | Tube 3 | | 96 |
| KKU-023 | Tube 1 | | 96.8 |
|  | Tube 2 | | 112 |
|  | Tube 3 | | 116 |
| MMNK-1 | Tube 1 | | 176 |
|  | Tube 2 | | 95.8 |
|  | Tube 3 | | 92.0 |

**S5 Table. Post-debranched mtDNA concentration.** These mtDNA concentrations were measured by Qubit fluorometer with dsDNA HS assay.

| Cell name | Replicate | dsDNA conc. (ng/µL) |
| --- | --- | --- |
| KKU-213A | Tube 1 | 482.4 |
|  | Tube 2 | 617.4 |
|  | Tube 3 | 507.6 |
| KKU-100 | Tube 1 | 633.6 |
|  | Tube 2 | 646.2 |
|  | Tube 3 | 487.8 |
| KKU-055 | Tube 1 | 489.6 |
|  | Tube 2 | 670.5 |
|  | Tube 3 | 518.4 |
| KKU-452 | Tube 1 | 612.0 |
|  | Tube 2 | 671.4 |
|  | Tube 3 | 428.4 |
| KKU-023 | Tube 1 | 406.8 |
|  | Tube 2 | 588.6 |
|  | Tube 3 | 894.6 |
| MMNK-1 | Tube 1 | 433.8 |
|  | Tube 2 | 727.2 |
|  | Tube 3 | 972.0 |

**S6 Table. INDELs of KKU-023 cell line.**

| Position | Ref | Variant | Heteroplasmy level (%) | INDEL | Maplocus | Category | OXPHOS  complex |
| --- | --- | --- | --- | --- | --- | --- | --- |
| 33 | CG | C | 9.9 | Deletion | MT-DLOOP2 | CR |  |
| 49 | AT | A | 6.5 | Deletion | MT-DLOOP2 | CR |  |
| 65 | T | TG | 4.6 | Insertion | MT-DLOOP2 | CR |  |
| 71 | GTA | G | 7.2 | Deletion | MT-DLOOP2 | CR |  |
| 302 | A | AC | 4.9 | Insertion | MT-DLOOP2 | CR |  |
| 310 | T | TC | 61.8 | Insertion | MT-DLOOP2 | CR |  |
| 356 | C | CA | 3.4 | Insertion | MT-DLOOP2 | CR |  |
| 432 | A | AC | 3.2 | Insertion | MT-DLOOP2 | CR |  |
| 455 | TC | T | 3.6 | Deletion | MT-DLOOP2 | CR |  |
| 460 | T | TC | 3.1 | Insertion | MT-DLOOP2 | CR |  |
| 493 | A | AC | 7.3 | Insertion | MT-DLOOP2 | CR |  |
| 499 | G | GC | 4.2 | Insertion | MT-DLOOP2 | CR |  |
| 513 | GCA | G | 95.2 | Deletion | MT-DLOOP2 | CR |  |
| 540 | AC | A | 5.5 | Deletion | MT-DLOOP2 | CR |  |
| 560 | CA | C | 9.7 | Deletion | MT-DLOOP2 | CR |  |
| 567 | AC | A | 13.4 | Deletion | MT-DLOOP2 | CR |  |
| 743 | CA | C | 2.4 | Deletion | *MT-RNR1* | rRNA |  |
| 801 | A | AC | 3.9 | Insertion | *MT-RNR1* | rRNA |  |
| 808 | CG | C | 5.4 | Deletion | *MT-RNR1* | rRNA |  |
| 949 | TA | T | 5.4 | Deletion | *MT-RNR1* | rRNA |  |
| 955 | AC | A | 7.3 | Deletion | *MT-RNR1* | rRNA |  |
| 961 | T | TC | 6.6 | Insertion | *MT-RNR1* | rRNA |  |
| 992 | T | TA | 3.9 | Insertion | *MT-RNR1* | rRNA |  |
| 1374 | A | AC | 3.1 | Insertion | *MT-RNR1* | rRNA |  |
| 1547 | TA | T | 4.6 | Deletion | *MT-RNR1* | rRNA |  |
| 1597 | CG | C | 4.3 | Deletion | *MT-RNR1* | rRNA |  |
| 1800 | GA | G | 5.5 | Deletion | *MT-RNR2* | rRNA |  |
| 1875 | CT | C | 2.7 | Deletion | *MT-RNR2* | rRNA |  |
| 1900 | AC | A | 19.7 | Deletion | *MT-RNR2* | rRNA |  |
| 1934 | T | TA | 3 | Insertion | *MT-RNR2* | rRNA |  |
| 2101 | C | CA | 3.7 | Insertion | *MT-RNR2* | rRNA |  |
| 2129 | GA | G | 4.5 | Deletion | *MT-RNR2* | rRNA |  |
| 2177 | T | TA | 4.9 | Insertion | *MT-RNR2* | rRNA |  |
| 2226 | T | TA | 3.2 | Insertion | *MT-RNR2* | rRNA |  |
| 2233 | TC | T | 3.2 | Deletion | *MT-RNR2* | rRNA |  |
| 2317 | G | GA | 2.6 | Insertion | *MT-RNR2* | rRNA |  |
| 2330 | T | TC | 3.1 | Insertion | *MT-RNR2* | rRNA |  |
| 2347 | CA | C | 5 | Deletion | *MT-RNR2* | rRNA |  |
| 2413 | CCCT | C | 2.7 | Deletion | *MT-RNR2* | rRNA |  |
| 2456 | T | TA | 8.3 | Insertion | *MT-RNR2* | rRNA |  |
| 2465 | TA | T | 4.2 | Deletion | *MT-RNR2* | rRNA |  |
| 2487 | AC | A | 8.5 | Deletion | *MT-RNR2* | rRNA |  |
| 2496 | G | GT | 2 | Insertion | *MT-RNR2* | rRNA |  |
| 2813 | TG | T | 7.3 | Deletion | *MT-RNR2* | rRNA |  |
| 3027 | TA | T | 5.1 | Deletion | *MT-RNR2* | rRNA |  |
| 3126 | CG | C | 2.2 | Deletion | *MT-RNR2* | rRNA |  |
| 3167 | TC | T | 4.3 | Deletion | *MT-RNR2* | rRNA |  |
| 3172 | CG | C | 2.9 | Deletion | *MT-RNR2* | rRNA |  |
| 3174 | T | TAA | 2.2 | Insertion | *MT-RNR2* | rRNA |  |
| 3244 | G | GC | 3.7 | Insertion | *MT-TL1* | tRNA |  |

| Position | Ref | Variant | Heteroplasmy level (%) | INDEL | Maplocus | Category | OXPHOS  complex |
| --- | --- | --- | --- | --- | --- | --- | --- |
| 3471 | CT | C | 3.5 | Deletion | *MT-ND1* | Coding | I |
| 3565 | A | AC | 2.8 | Insertion | *MT-ND1* | Coding | I |
| 3572 | T | TC | 9.7 | Insertion | *MT-ND1* | Coding | I |
| 3584 | AC | A | 5.9 | Deletion | *MT-ND1* | Coding | I |
| 4057 | TC | T | 5.7 | Deletion | *MT-ND1* | Coding | I |
| 4136 | AC | A | 16.5 | Deletion | *MT-ND1* | Coding | I |
| 4248 | TC | T | 3.7 | Deletion | *MT-ND1* | Coding | I |
| 4435 | AC | A | 4.5 | Deletion | *MT-TM* | tRNA |  |
| 4460 | T | TC | 5.2 | Insertion | *MT-TM* | tRNA |  |
| 4794 | GC | G | 6.2 | Deletion | *MT-ND2* | Coding | I |
| 4869 | CA | C | 5.6 | Deletion | *MT-ND2* | Coding | I |
| 4878 | GC | G | 11.7 | Deletion | *MT-ND2* | Coding | I |
| 5217 | T | TC | 2.2 | Insertion | *MT-ND2* | Coding | I |
| 5220 | C | CT | 2.4 | Insertion | *MT-ND2* | Coding | I |
| 5231 | GC | G | 12.7 | Deletion | *MT-ND2* | Coding | I |
| 5281 | C | CA | 2 | Insertion | *MT-ND2* | Coding | I |
| 5487 | TC | T | 2.4 | Deletion | *MT-ND2* | Coding | I |
| 5491 | C | CT | 2 | Insertion | *MT-ND2* | Coding | I |
| 5507 | CT | C | 2.6 | Deletion | *MT-ND2* | Coding | I |
| 5763 | GC | G | 12 | Deletion | *MT-TC* | tRNA |  |
| 5829 | TA | T | 7.5 | Deletion | *MT-TY* | tRNA |  |
| 6152 | TC | T | 8.5 | Deletion | *MT-CO1* | Coding | IV |
| 6223 | C | CCT | 3.1 | Insertion | *MT-CO1* | Coding | IV |
| 6446 | G | GC | 3.3 | Insertion | *MT-CO1* | Coding | IV |
| 6565 | AC | A | 5 | Deletion | *MT-CO1* | Coding | IV |
| 6572 | CGGA | C | 15.1 | Deletion | *MT-CO1* | Coding | IV |
| 6583 | A | AC | 4.3 | Insertion | *MT-CO1* | Coding | IV |
| 6611 | AT | A | 5.8 | Deletion | *MT-CO1* | Coding | IV |
| 6691 | G | GA | 8.1 | Insertion | *MT-CO1* | Coding | IV |
| 6942 | CT | C | 7.8 | Deletion | *MT-CO1* | Coding | IV |
| 7094 | TC | T | 9.3 | Deletion | *MT-CO1* | Coding | IV |
| 7179 | TTC | T | 2.8 | Deletion | *MT-CO1* | Coding | IV |
| 7211 | GC | G | 9.2 | Deletion | *MT-CO1* | Coding | IV |
| 7231 | AC | A | 4.2 | Deletion | *MT-CO1* | Coding | IV |
| 7341 | C | CG | 6.3 | Insertion | *MT-CO1* | Coding | IV |
| 7396 | GC | G | 15.1 | Deletion | *MT-CO1* | Coding | IV |
| 7446 | C | CA | 3.7 | Insertion | *MT-TS1* | tRNA |  |
| 7465 | AC | A | 6.6 | Deletion | *MT-TS1* | tRNA |  |
| 7514 | CA | C | 8.8 | Deletion | *MT-TS1* | tRNA |  |
| 7625 | TC | T | 4.2 | Deletion | *MT-CO2* | Coding | IV |
| 7709 | CT | C | 17.8 | Deletion | *MT-CO2* | Coding | IV |
| 7818 | TC | T | 7.8 | Deletion | *MT-CO2* | Coding | IV |
| 7842 | TA | T | 2.7 | Deletion | *MT-CO2* | Coding | IV |
| 7954 | T | TC | 6.3 | Insertion | *MT-CO2* | Coding | IV |
| 8027 | GC | G | 5.4 | Deletion | *MT-CO2* | Coding | IV |
| 8079 | TC | T | 6.9 | Deletion | *MT-CO2* | Coding | IV |
| 8151 | CG | C | 11.4 | Deletion | *MT-CO2* | Coding | IV |
| 8232 | TA | T | 5.4 | Deletion | *MT-CO2* | Coding | IV |
| 8280 | AC | A | 5.7 | Deletion | NA | - |  |
| 8354 | CT | C | 7.4 | Deletion | *MT-TK* | tRNA |  |

**S6 Table. INDELs of KKU-023 cell line (cont.).**

| Position | Ref | Variant | Heteroplasmy level (%) | INDEL | Maplocus | Category | OXPHOS  complex |
| --- | --- | --- | --- | --- | --- | --- | --- |
| 8368 | G | GC | 3 | Insertion | *MT-AYP8* | Coding | V |
| 8449 | AT | A | 5.8 | Deletion | *MT-AYP8* | Coding | V |
| 8490 | TA | T | 4.7 | Deletion | *MT-AYP8* | Coding | V |
| 8604 | TC | T | 13.6 | Deletion | *MT-ATP6* | Coding | V |
| 8939 | T | TC | 2.3 | Insertion | *MT-ATP6* | Coding | V |
| 9449 | CG | C | 6.3 | Deletion | *MT-CO3* | Coding | IV |
| 9477 | G | GT | 2.1 | Insertion | *MT-CO3* | Coding | IV |
| 9506 | C | CT | 3.1 | Insertion | *MT-CO3* | Coding | IV |
| 9531 | ACC | A | 5.7 | Deletion | *MT-CO3* | Coding | IV |
| 9531 | A | AC | 5.8 | Insertion | *MT-CO3* | Coding | IV |
| 9721 | AT | A | 6.2 | Deletion | *MT-CO3* | Coding | IV |
| 9794 | AT | A | 7.1 | Deletion | *MT-CO3* | Coding | IV |
| 9994 | CT | C | 2.5 | Deletion | *MT-TG* | tRNA |  |
| 10047 | C | CA | 3.5 | Insertion | *MT-TG* | tRNA |  |
| 10191 | T | TC | 4.3 | Insertion | *MT-ND3* | Coding | I |
| 10204 | T | TC | 3.3 | Insertion | *MT-ND3* | Coding | I |
| 10241 | C | CT | 2.1 | Insertion | *MT-ND3* | Coding | I |
| 10272 | CT | C | 10.5 | Deletion | *MT-ND3* | Coding | I |
| 10357 | T | TA | 4.9 | Insertion | *MT-ND3* | Coding | I |
| 10380 | CA | C | 2.1 | Deletion | *MT-ND3* | Coding | I |
| 10813 | C | CA | 2.1 | Insertion | *MT-ND4* | Coding | I |
| 10879 | AT | A | 5.6 | Deletion | *MT-ND4* | Coding | I |
| 10916 | T | TC | 2.6 | Insertion | *MT-ND4* | Coding | I |
| 10935 | AC | A | 15.9 | Deletion | *MT-ND4* | Coding | I |
| 10978 | AC | A | 2.1 | Deletion | *MT-ND4* | Coding | I |
| 11031 | GA | G | 5.9 | Deletion | *MT-ND4* | Coding | I |
| 11124 | TC | T | 3.5 | Deletion | *MT-ND4* | Coding | I |
| 11139 | T | TC | 4.1 | Insertion | *MT-ND4* | Coding | I |
| 11233 | T | TC | 4.2 | Insertion | *MT-ND4* | Coding | I |
| 11280 | TA | T | 5.8 | Deletion | *MT-ND4* | Coding | I |
| 11426 | G | GC | 2.2 | Insertion | *MT-ND4* | Coding | I |
| 11466 | T | TA | 3.4 | Insertion | *MT-ND4* | Coding | I |
| 11625 | CT | C | 6.4 | Deletion | *MT-ND4* | Coding | I |
| 11788 | CCT | C | 3 | Deletion | *MT-ND4* | Coding | I |
| 11826 | CT | C | 2.1 | Deletion | *MT-ND4* | Coding | I |
| 11866 | AC | A | 16.3 | Deletion | *MT-ND4* | Coding | I |
| 12005 | TG | T | 2.3 | Deletion | *MT-ND4* | Coding | I |
| 12083 | TC | T | 34.2 | Deletion | *MT-ND4* | Coding | I |
| 12135 | CT | C | 7.3 | Deletion | *MT-ND4* | Coding | I |
| 12236 | GC | G | 5.7 | Deletion | *MT-TS2* | tRNA |  |
| 12305 | CA | C | 3.3 | Deletion | *MT-TL2* | tRNA |  |
| 12329 | T | TA | 2.2 | Insertion | *MT-TL2* | tRNA |  |
| 12384 | T | TC | 2 | Insertion | *MT-ND5* | Coding | I |
| 12417 | C | CA | 6.4 | Insertion | *MT-ND5* | Coding | I |
| 12565 | T | TC | 2.4 | Insertion | *MT-ND5* | Coding | I |
| 13127 | AC | A | 16.5 | Deletion | *MT-ND5* | Coding | I |
| 13196 | TCG | T | 2.3 | Deletion | *MT-ND5* | Coding | I |
| 13230 | CAAA | C | 4.5 | Deletion | *MT-ND5* | Coding | I |
| 13230 | C | CA | 3.6 | Insertion | *MT-ND5* | Coding | I |
| 13365 | CG | C | 4.5 | Deletion | *MT-ND5* | Coding | I |

**S6 Table. INDELs of KKU-023 cell line (cont.).**

| Position | Ref | Variant | Heteroplasmy level (%) | INDEL | Maplocus | Category | OXPHOS  complex |
| --- | --- | --- | --- | --- | --- | --- | --- |
| 13370 | C | CT | 4.5 | Insertion | *MT-ND5* | Coding | I |
| 13406 | G | GA | 2.3 | Insertion | *MT-ND5* | Coding | I |
| 13585 | T | TC | 3.4 | Insertion | *MT-ND5* | Coding | I |
| 13646 | T | TC | 7.8 | Insertion | *MT-ND5* | Coding | I |
| 13753 | TC | T | 5.5 | Deletion | *MT-ND5* | Coding | I |
| 13766 | CCT | C | 2.9 | Deletion | *MT-ND5* | Coding | I |
| 13820 | T | TC | 4.7 | Insertion | *MT-ND5* | Coding | I |
| 13980 | GC | G | 9.1 | Deletion | *MT-ND5* | Coding | I |
| 14105 | C | CT | 6.2 | Insertion | *MT-ND5* | Coding | I |
| 14154 | TC | T | 10.5 | Deletion | *MT-ND6* | Coding | I |
| 14158 | CCG | C | 4.8 | Deletion | *MT-ND6* | Coding | I |
| 14264 | T | TC | 3 | Insertion | *MT-ND6* | Coding | I |
| 14339 | AC | A | 4.2 | Deletion | *MT-ND6* | Coding | I |
| 14395 | T | TA | 3.6 | Insertion | *MT-ND6* | Coding | I |
| 14488 | TC | T | 4.3 | Deletion | *MT-ND6* | Coding | I |
| 14503 | TA | T | 5.3 | Deletion | *MT-ND6* | Coding | I |
| 14530 | T | TC | 6 | Insertion | *MT-ND6* | Coding | I |
| 14808 | T | TC | 4.8 | Insertion | *MT-CYB* | Coding | III |
| 14813 | AC | A | 8.6 | Deletion | *MT-CYB* | Coding | III |
| 15262 | T | TC | 4.3 | Insertion | *MT-CYB* | Coding | III |
| 15303 | CCT | C | 2 | Deletion | *MT-CYB* | Coding | III |
| 15536 | A | AC | 2.5 | Insertion | *MT-CYB* | Coding | III |
| 15541 | T | TC | 6.7 | Insertion | *MT-CYB* | Coding | III |
| 15590 | CG | C | 5.1 | Deletion | *MT-CYB* | Coding | III |
| 15593 | T | TC | 2.5 | Insertion | *MT-CYB* | Coding | III |
| 15939 | CT | C | 4.9 | Deletion | *MT-TT* | tRNA |  |
| 15961 | G | GA | 4.9 | Insertion | *MT-TP* | tRNA |  |
| 16042 | GAT | G | 5.4 | Deletion | MT-DLOOP1 | CR |  |
| 16289 | A | AC | 3.2 | Insertion | MT-DLOOP1 | CR |  |
| 16374 | AC | A | 3.7 | Deletion | MT-DLOOP1 | CR |  |
| 16469 | TG | T | 5 | Deletion | MT-DLOOP1 | CR |  |

**S7 Table. INDELs of KKU-055 cell line.**

| Position | Ref | Variant | Heteroplasmy level (%) | INDEL | Maplocus | Category | OXPHOS  complex |
| --- | --- | --- | --- | --- | --- | --- | --- |
| 33 | CG | C | 13.2 | Deletion | MT-DLOOP2 | CR |  |
| 49 | AT | A | 7.5 | Deletion | MT-DLOOP2 | CR |  |
| 65 | T | TG | 2.8 | Insertion | MT-DLOOP2 | CR |  |
| 71 | GTA | G | 6.7 | Deletion | MT-DLOOP2 | CR |  |
| 247 | GA | G | 97.7 | Deletion | MT-DLOOP2 | CR |  |
| 302 | A | AC | 7.4 | Insertion | MT-DLOOP2 | CR |  |
| 302 | A | ACCC | 2.3 | Insertion | MT-DLOOP2 | CR |  |
| 310 | T | TC | 62.3 | Insertion | MT-DLOOP2 | CR |  |
| 356 | CA | C | 2.1 | Deletion | MT-DLOOP2 | CR |  |
| 432 | AC | A | 10.1 | Deletion | MT-DLOOP2 | CR |  |
| 455 | T | TC | 2.3 | Insertion | MT-DLOOP2 | CR |  |
| 460 | TC | T | 4.3 | Deletion | MT-DLOOP2 | CR |  |
| 460 | T | TC | 2.3 | Insertion | MT-DLOOP2 | CR |  |
| 513 | GCA | G | 94 | Deletion | MT-DLOOP2 | CR |  |
| 540 | A | AC | 4.3 | Insertion | MT-DLOOP2 | CR |  |
| 567 | AC | A | 12.1 | Deletion | MT-DLOOP2 | CR |  |
| 743 | CA | C | 2.1 | Deletion | *MT-RNR1* | rRNA |  |
| 747 | A | AG | 2.3 | Insertion | *MT-RNR1* | rRNA |  |
| 801 | AC | A | 3.1 | Deletion | *MT-RNR1* | rRNA |  |
| 808 | CG | C | 5.9 | Deletion | *MT-RNR1* | rRNA |  |
| 955 | AC | A | 7.4 | Deletion | *MT-RNR1* | rRNA |  |
| 961 | TC | T | 9.8 | Deletion | *MT-RNR1* | rRNA |  |
| 961 | T | TC | 8.7 | Insertion | *MT-RNR1* | rRNA |  |
| 992 | T | TA | 2.8 | Insertion | *MT-RNR1* | rRNA |  |
| 1165 | C | CA | 2.5 | Insertion | *MT-RNR1* | rRNA |  |
| 1367 | A | AT | 2.8 | Insertion | *MT-RNR1* | rRNA |  |
| 1374 | A | AC | 4.8 | Insertion | *MT-RNR1* | rRNA |  |
| 1547 | TA | T | 4.3 | Deletion | *MT-RNR1* | rRNA |  |
| 1597 | CG | C | 8.4 | Deletion | *MT-RNR1* | rRNA |  |
| 1681 | G | GC | 3.1 | Insertion | *MT-RNR2* | rRNA |  |
| 1790 | A | AG | 2.8 | Insertion | *MT-RNR2* | rRNA |  |
| 1800 | GA | G | 5 | Deletion | *MT-RNR2* | rRNA |  |
| 1875 | CT | C | 2.7 | Deletion | *MT-RNR2* | rRNA |  |
| 1900 | AC | A | 9.9 | Deletion | *MT-RNR2* | rRNA |  |
| 1934 | TA | T | 4.9 | Deletion | *MT-RNR2* | rRNA |  |
| 1945 | A | AC | 2 | Insertion | *MT-RNR2* | rRNA |  |
| 2101 | C | CA | 2.3 | Insertion | *MT-RNR2* | rRNA |  |
| 2129 | GA | G | 4.5 | Deletion | *MT-RNR2* | rRNA |  |
| 2177 | TA | T | 11.6 | Deletion | *MT-RNR2* | rRNA |  |
| 2226 | T | TA | 3.2 | Insertion | *MT-RNR2* | rRNA |  |
| 2233 | TC | T | 2.6 | Deletion | *MT-RNR2* | rRNA |  |
| 2347 | CA | C | 7.3 | Deletion | *MT-RNR2* | rRNA |  |
| 2413 | CCCT | C | 2.4 | Deletion | *MT-RNR2* | rRNA |  |
| 2456 | T | TA | 5.7 | Insertion | *MT-RNR2* | rRNA |  |
| 2465 | TA | T | 4.7 | Deletion | *MT-RNR2* | rRNA |  |
| 2487 | AC | A | 14.4 | Deletion | *MT-RNR2* | rRNA |  |
| 2496 | G | GT | 3.3 | Insertion | *MT-RNR2* | rRNA |  |
| 2800 | TA | T | 4.4 | Deletion | *MT-RNR2* | rRNA |  |
| 2813 | TG | T | 5.3 | Deletion | *MT-RNR2* | rRNA |  |
| 3126 | CG | C | 5.5 | Deletion | *MT-RNR2* | rRNA |  |

**S7 Table. INDELs of KKU-055 cell line (cont.).**

| Position | Ref | Variant | Heteroplasmy level (%) | INDEL | Maplocus | Category | OXPHOS  complex |
| --- | --- | --- | --- | --- | --- | --- | --- |
| 3167 | TC | T | 8.8 | Deletion | *MT-RNR2* | rRNA |  |
| 3174 | T | TA | 3.7 | Insertion | *MT-RNR2* | rRNA |  |
| 3209 | A | AC | 2.4 | Insertion | *MT-RNR2* | rRNA |  |
| 3244 | G | GC | 3.8 | Insertion | *MT-TL1* | tRNA |  |
| 3483 | G | GC | 4 | Insertion | *MT-ND1* | Coding | I |
| 3565 | AC | A | 2.4 | Deletion | *MT-ND1* | Coding | I |
| 3572 | T | TC | 9 | Insertion | *MT-ND1* | Coding | I |
| 3584 | AC | A | 4 | Deletion | *MT-ND1* | Coding | I |
| 3783 | CT | C | 9.3 | Deletion | *MT-ND1* | Coding | I |
| 4057 | TC | T | 6.8 | Deletion | *MT-ND1* | Coding | I |
| 4136 | AC | A | 17.1 | Deletion | *MT-ND1* | Coding | I |
| 4248 | T | TC | 2.5 | Insertion | *MT-ND1* | Coding | I |
| 4325 | A | AT | 2.8 | Insertion | *MT-TI* | tRNA |  |
| 4435 | A | AC | 3.4 | Insertion | *MT-TM* | tRNA |  |
| 4439 | CG | C | 3.9 | Deletion | *MT-TM* | tRNA |  |
| 4460 | T | TC | 7.5 | Insertion | *MT-TM* | tRNA |  |
| 4585 | CT | C | 4.4 | Deletion | *MT-ND2* | Coding | I |
| 4794 | G | GC | 4 | Insertion | *MT-ND2* | Coding | I |
| 4799 | C | CT | 2.1 | Insertion | *MT-ND2* | Coding | I |
| 4869 | CA | C | 5.6 | Deletion | *MT-ND2* | Coding | I |
| 4878 | GC | G | 13 | Deletion | *MT-ND2* | Coding | I |
| 5195 | CT | C | 5.5 | Deletion | *MT-ND2* | Coding | I |
| 5231 | GC | G | 14 | Deletion | *MT-ND2* | Coding | I |
| 5383 | T | TC | 2.3 | Insertion | *MT-ND2* | Coding | I |
| 5446 | T | TC | 2.1 | Insertion | *MT-ND2* | Coding | I |
| 5487 | T | TC | 2.3 | Insertion | *MT-ND2* | Coding | I |
| 5490 | CCT | C | 4.8 | Deletion | *MT-ND2* | Coding | I |
| 5743 | CG | C | 12.7 | Deletion | NA | - |  |
| 5746 | GA | G | 6 | Deletion | NA | - |  |
| 5756 | GGGAGA | G | 10.8 | Deletion | NA | - |  |
| 5763 | GC | G | 18 | Deletion | *MT-TC* | tRNA |  |
| 6152 | TC | T | 11.4 | Deletion | *MT-CO1* | Coding | IV |
| 6185 | TC | T | 14.9 | Deletion | *MT-CO1* | Coding | IV |
| 6223 | CCT | C | 4.5 | Deletion | *MT-CO1* | Coding | IV |
| 6312 | TC | T | 7.6 | Deletion | *MT-CO1* | Coding | IV |
| 6446 | G | GC | 5.6 | Insertion | *MT-CO1* | Coding | IV |
| 6583 | A | AC | 8.5 | Insertion | *MT-CO1* | Coding | IV |
| 6691 | G | GA | 6.3 | Insertion | *MT-CO1* | Coding | IV |
| 6942 | CT | C | 11 | Deletion | *MT-CO1* | Coding | IV |
| 7091 | A | AT | 2.4 | Insertion | *MT-CO1* | Coding | IV |
| 7094 | TC | T | 12.5 | Deletion | *MT-CO1* | Coding | IV |
| 7180 | TC | T | 13.1 | Deletion | *MT-CO1* | Coding | IV |
| 7211 | GC | G | 10.1 | Deletion | *MT-CO1* | Coding | IV |
| 7231 | AC | A | 5 | Deletion | *MT-CO1* | Coding | IV |
| 7341 | C | CG | 12.4 | Insertion | *MT-CO1* | Coding | IV |
| 7396 | GC | G | 21.6 | Deletion | *MT-CO1* | Coding | IV |
| 7446 | C | CA | 2.1 | Insertion | *MT-TS1* | tRNA |  |
| 7465 | A | AC | 3.1 | Insertion | *MT-TS1* | tRNA |  |
| 7514 | C | CA | 2 | Insertion | *MT-TS1* | tRNA |  |

**S7 Table. INDELs of KKU-055 cell line (cont.).**

| Position | Ref | Variant | Heteroplasmy level (%) | INDEL | Maplocus | Category | OXPHOS  complex |
| --- | --- | --- | --- | --- | --- | --- | --- |
| 7625 | TC | T | 4.3 | Deletion | *MT-CO2* | Coding | IV |
| 7709 | C | CT | 5.1 | Insertion | *MT-CO2* | Coding | IV |
| 7842 | TA | T | 2.3 | Deletion | *MT-CO2* | Coding | IV |
| 7954 | T | TC | 2.9 | Insertion | *MT-CO2* | Coding | IV |
| 8006 | CG | C | 7.8 | Deletion | *MT-CO2* | Coding | IV |
| 8016 | T | TC | 4.5 | Insertion | *MT-CO2* | Coding | IV |
| 8079 | TC | T | 11 | Deletion | *MT-CO2* | Coding | IV |
| 8094 | T | TA | 4.4 | Insertion | *MT-CO2* | Coding | IV |
| 8151 | CG | C | 9.6 | Deletion | *MT-CO2* | Coding | IV |
| 8232 | TA | T | 7.2 | Deletion | *MT-CO2* | Coding | IV |
| 8271 | AC | A | 6.2 | Deletion | NA | - |  |
| 8354 | CT | C | 6.9 | Deletion | *MT-TK* | tRNA |  |
| 8368 | G | GC | 7.8 | Insertion | *MT-AYP8* | Coding | V |
| 8604 | TC | T | 16.4 | Deletion | *MT-ATP6* | Coding | V |
| 8902 | G | GC | 2.2 | Insertion | *MT-ATP6* | Coding | V |
| 8931 | AC | A | 3.1 | Deletion | *MT-ATP6* | Coding | V |
| 8939 | T | TC | 2.7 | Insertion | *MT-ATP6* | Coding | V |
| 9291 | T | TC | 3.9 | Insertion | *MT-CO3* | Coding | IV |
| 9477 | GT | G | 2.5 | Deletion | *MT-CO3* | Coding | IV |
| 9531 | ACC | A | 4.2 | Deletion | *MT-CO3* | Coding | IV |
| 9531 | A | AC | 3.2 | Insertion | *MT-CO3* | Coding | IV |
| 9537 | C | CA | 2.8 | Insertion | *MT-CO3* | Coding | IV |
| 9663 | GA | G | 8 | Deletion | *MT-CO3* | Coding | IV |
| 9721 | AT | A | 6.5 | Deletion | *MT-CO3* | Coding | IV |
| 9794 | AT | A | 6.2 | Deletion | *MT-CO3* | Coding | IV |
| 9994 | C | CT | 2.2 | Insertion | *MT-TG* | tRNA |  |
| 10047 | C | CA | 7 | Insertion | *MT-TG* | tRNA |  |
| 10089 | A | AC | 3 | Insertion | *MT-ND3* | Coding | I |
| 10191 | T | TC | 4.4 | Insertion | *MT-ND3* | Coding | I |
| 10207 | C | CT | 3.4 | Insertion | *MT-ND3* | Coding | I |
| 10244 | C | CT | 2.8 | Insertion | *MT-ND3* | Coding | I |
| 10272 | CT | C | 10.1 | Deletion | *MT-ND3* | Coding | I |
| 10357 | T | TA | 3.6 | Insertion | *MT-ND3* | Coding | I |
| 10380 | CA | C | 2.5 | Deletion | *MT-ND3* | Coding | I |
| 10631 | C | CT | 4.6 | Insertion | *MT-ND4L* | Coding | I |
| 10687 | TG | T | 7 | Deletion | *MT-ND4L* | Coding | I |
| 10879 | AT | A | 3.1 | Deletion | *MT-ND4* | Coding | I |
| 10916 | T | TC | 2.4 | Insertion | *MT-ND4* | Coding | I |
| 10935 | AC | A | 11 | Deletion | *MT-ND4* | Coding | I |
| 11031 | GA | G | 9.1 | Deletion | *MT-ND4* | Coding | I |
| 11122 | C | CT | 2.5 | Insertion | *MT-ND4* | Coding | I |
| 11139 | T | TC | 4 | Insertion | *MT-ND4* | Coding | I |
| 11233 | T | TC | 3.9 | Insertion | *MT-ND4* | Coding | I |
| 11280 | TA | T | 5 | Deletion | *MT-ND4* | Coding | I |
| 11384 | C | CT | 3.7 | Insertion | *MT-ND4* | Coding | I |
| 11426 | GC | G | 2.8 | Deletion | *MT-ND4* | Coding | I |
| 11466 | T | TA | 3.5 | Insertion | *MT-ND4* | Coding | I |
| 11511 | AC | A | 3.4 | Deletion | *MT-ND4* | Coding | I |
| 11535 | AC | A | 2.7 | Deletion | *MT-ND4* | Coding | I |
| 11604 | TA | T | 2.2 | Deletion | *MT-ND4* | Coding | I |

**S7 Table. INDELs of KKU-055 cell line (cont.).**

| Position | Ref | Variant | Heteroplasmy level (%) | INDEL | Maplocus | Category | OXPHOS  complex |
| --- | --- | --- | --- | --- | --- | --- | --- |
| 11826 | CT | C | 2.2 | Deletion | *MT-ND4* | Coding | I |
| 11866 | A | AC | 3.9 | Insertion | *MT-ND4* | Coding | I |
| 12005 | TG | T | 2.4 | Deletion | *MT-ND4* | Coding | I |
| 12053 | C | CA | 2.4 | Insertion | *MT-ND4* | Coding | I |
| 12083 | TC | T | 30.5 | Deletion | *MT-ND4* | Coding | I |
| 12236 | GC | G | 7.4 | Deletion | *MT-TS2* | tRNA |  |
| 12305 | CA | C | 5.2 | Deletion | *MT-TL2* | tRNA |  |
| 12384 | TC | T | 21.6 | Deletion | *MT-ND5* | Coding | I |
| 12417 | C | CA | 6.2 | Insertion | *MT-ND5* | Coding | I |
| 12431 | AC | A | 3.8 | Deletion | *MT-ND5* | Coding | I |
| 12482 | TC | T | 2.9 | Deletion | *MT-ND5* | Coding | I |
| 12565 | T | TC | 3.3 | Insertion | *MT-ND5* | Coding | I |
| 12638 | TA | T | 2.9 | Deletion | *MT-ND5* | Coding | I |
| 13034 | TC | T | 5.5 | Deletion | *MT-ND5* | Coding | I |
| 13127 | AC | A | 10.7 | Deletion | *MT-ND5* | Coding | I |
| 13145 | G | GC | 2.5 | Insertion | *MT-ND5* | Coding | I |
| 13230 | CAAA | C | 4.8 | Deletion | *MT-ND5* | Coding | I |
| 13230 | C | CA | 3.3 | Insertion | *MT-ND5* | Coding | I |
| 13365 | CG | C | 4.7 | Deletion | *MT-ND5* | Coding | I |
| 13370 | C | CT | 2.3 | Insertion | *MT-ND5* | Coding | I |
| 13646 | T | TC | 5.6 | Insertion | *MT-ND5* | Coding | I |
| 13753 | TC | T | 8.5 | Deletion | *MT-ND5* | Coding | I |
| 13762 | TC | T | 6.3 | Deletion | *MT-ND5* | Coding | I |
| 13842 | AG | A | 3 | Deletion | *MT-ND5* | Coding | I |
| 13980 | GC | G | 13.1 | Deletion | *MT-ND5* | Coding | I |
| 14109 | CT | C | 4.9 | Deletion | *MT-ND5* | Coding | I |
| 14154 | TC | T | 19.1 | Deletion | *MT-ND6* | Coding | I |
| 14339 | AC | A | 4.4 | Deletion | *MT-ND6* | Coding | I |
| 14395 | T | TA | 3 | Insertion | *MT-ND6* | Coding | I |
| 14503 | TA | T | 3.5 | Deletion | *MT-ND6* | Coding | I |
| 14530 | T | TC | 2.6 | Insertion | *MT-ND6* | Coding | I |
| 14808 | T | TC | 2.5 | Insertion | *MT-CYB* | Coding | III |
| 14813 | AC | A | 10.8 | Deletion | *MT-CYB* | Coding | III |
| 15221 | GA | G | 2.5 | Deletion | *MT-CYB* | Coding | III |
| 15224 | C | CT | 2.8 | Insertion | *MT-CYB* | Coding | III |
| 15262 | T | TC | 3.8 | Insertion | *MT-CYB* | Coding | III |
| 15349 | CG | C | 4.2 | Deletion | *MT-CYB* | Coding | III |
| 15366 | ACC | A | 4.6 | Deletion | *MT-CYB* | Coding | III |
| 15541 | T | TC | 3.3 | Insertion | *MT-CYB* | Coding | III |
| 15593 | T | TC | 7.8 | Insertion | *MT-CYB* | Coding | III |
| 15874 | AC | A | 2.8 | Deletion | *MT-CYB* | Coding | III |
| 15939 | CT | C | 3.1 | Deletion | *MT-TT* | tRNA |  |
| 15961 | G | GA | 5.5 | Insertion | *MT-TP* | tRNA |  |
| 16189 | T | TC | 7.6 | Insertion | MT-DLOOP1 | CR |  |
| 16289 | A | AC | 3.1 | Insertion | MT-DLOOP1 | CR |  |
| 16431 | CA | C | 6 | Deletion | MT-DLOOP1 | CR |  |
| 16469 | TG | T | 4.3 | Deletion | MT-DLOOP1 | CR |  |

**S8 Table. INDELs of KKU-100 cell line.**

| Position | Ref | Variant | Heteroplasmy level (%) | INDEL | Maplocus | Category | OXPHOS  complex |
| --- | --- | --- | --- | --- | --- | --- | --- |
| 33 | CG | C | 10.3 | Deletion | MT-DLOOP2 | CR |  |
| 49 | AT | A | 4.9 | Deletion | MT-DLOOP2 | CR |  |
| 64 | CT | C | 2.1 | Deletion | MT-DLOOP2 | CR |  |
| 65 | TGG | T | 4.7 | Deletion | MT-DLOOP2 | CR |  |
| 65 | T | TG | 2.5 | Insertion | MT-DLOOP2 | CR |  |
| 285 | C | CA | 2.3 | Insertion | MT-DLOOP2 | CR |  |
| 302 | A | AC | 48.4 | Insertion | MT-DLOOP2 | CR |  |
| 302 | A | ACCC | 8.5 | Insertion | MT-DLOOP2 | CR |  |
| 310 | T | TC | 72.8 | Insertion | MT-DLOOP2 | CR |  |
| 356 | CA | C | 3.8 | Deletion | MT-DLOOP2 | CR |  |
| 432 | A | AC | 4.4 | Insertion | MT-DLOOP2 | CR |  |
| 455 | T | TC | 4.7 | Insertion | MT-DLOOP2 | CR |  |
| 493 | A | AC | 2.9 | Insertion | MT-DLOOP2 | CR |  |
| 513 | G | GCA | 2.4 | Insertion | MT-DLOOP2 | CR |  |
| 540 | A | AC | 2.8 | Insertion | MT-DLOOP2 | CR |  |
| 560 | CA | C | 11 | Deletion | MT-DLOOP2 | CR |  |
| 567 | A | AC | 2.1 | Insertion | MT-DLOOP2 | CR |  |
| 597 | C | CA | 3 | Insertion | *MT-TF* | tRNA |  |
| 747 | A | AG | 2.2 | Insertion | *MT-RNR1* | rRNA |  |
| 955 | AC | A | 6.1 | Deletion | *MT-RNR1* | rRNA |  |
| 961 | T | TC | 8.5 | Insertion | *MT-RNR1* | rRNA |  |
| 992 | T | TA | 2.9 | Insertion | *MT-RNR1* | rRNA |  |
| 1165 | C | CA | 3.2 | Insertion | *MT-RNR1* | rRNA |  |
| 1374 | A | AC | 5.9 | Insertion | *MT-RNR1* | rRNA |  |
| 1547 | TA | T | 4.7 | Deletion | *MT-RNR1* | rRNA |  |
| 1597 | CG | C | 7.5 | Deletion | *MT-RNR1* | rRNA |  |
| 1900 | AC | A | 21.7 | Deletion | *MT-RNR2* | rRNA |  |
| 1934 | T | TA | 2.8 | Insertion | *MT-RNR2* | rRNA |  |
| 2116 | C | CT | 2 | Insertion | *MT-RNR2* | rRNA |  |
| 2129 | GA | G | 2.8 | Deletion | *MT-RNR2* | rRNA |  |
| 2177 | T | TA | 4.6 | Insertion | *MT-RNR2* | rRNA |  |
| 2226 | T | TA | 3.3 | Insertion | *MT-RNR2* | rRNA |  |
| 2456 | T | TA | 5.9 | Insertion | *MT-RNR2* | rRNA |  |
| 2465 | TA | T | 4.7 | Deletion | *MT-RNR2* | rRNA |  |
| 2487 | AC | A | 11.2 | Deletion | *MT-RNR2* | rRNA |  |
| 2496 | GT | G | 2.5 | Deletion | *MT-RNR2* | rRNA |  |
| 2502 | CA | C | 2.8 | Deletion | *MT-RNR2* | rRNA |  |
| 2800 | TA | T | 3.4 | Deletion | *MT-RNR2* | rRNA |  |
| 2813 | TG | T | 6.3 | Deletion | *MT-RNR2* | rRNA |  |
| 3027 | TA | T | 4 | Deletion | *MT-RNR2* | rRNA |  |
| 3040 | G | GT | 2.1 | Insertion | *MT-RNR2* | rRNA |  |
| 3126 | CG | C | 3.5 | Deletion | *MT-RNR2* | rRNA |  |
| 3167 | TC | T | 7.9 | Deletion | *MT-RNR2* | rRNA |  |
| 3174 | T | TAA | 2.5 | Insertion | *MT-RNR2* | rRNA |  |
| 3565 | AC | A | 3.9 | Deletion | *MT-ND1* | Coding | I |
| 3572 | T | TC | 8.1 | Insertion | *MT-ND1* | Coding | I |
| 3584 | AC | A | 5.6 | Deletion | *MT-ND1* | Coding | I |
| 4057 | TC | T | 4.4 | Deletion | *MT-ND1* | Coding | I |
| 4121 | GA | G | 2.4 | Deletion | *MT-ND1* | Coding | I |
| 4136 | AC | A | 16.5 | Deletion | *MT-ND1* | Coding | I |

**S8 Table. INDELs of KKU-100 cell line (cont.)**

| Position | Ref | Variant | Heteroplasmy level (%) | INDEL | Maplocus | Category | OXPHOS  complex |
| --- | --- | --- | --- | --- | --- | --- | --- |
| 4248 | T | TC | 2.6 | Insertion | *MT-ND1* | Coding | I |
| 4261 | TA | T | 2 | Deletion | *MT-ND1* | Coding | I |
| 4325 | A | AT | 2.1 | Insertion | *MT-TI* | tRNA |  |
| 4425 | TC | T | 2.7 | Deletion | *MT-TM* | tRNA |  |
| 4439 | CG | C | 5.5 | Deletion | *MT-TM* | tRNA |  |
| 4460 | T | TC | 3 | Insertion | *MT-TM* | tRNA |  |
| 4604 | C | CA | 3 | Insertion | *MT-ND2* | Coding | I |
| 4794 | GC | G | 6.4 | Deletion | *MT-ND2* | Coding | I |
| 4869 | CA | C | 6.3 | Deletion | *MT-ND2* | Coding | I |
| 4878 | GC | G | 12.6 | Deletion | *MT-ND2* | Coding | I |
| 5217 | T | TC | 7.3 | Insertion | *MT-ND2* | Coding | I |
| 5231 | GC | G | 15.8 | Deletion | *MT-ND2* | Coding | I |
| 5281 | C | CA | 2.7 | Insertion | *MT-ND2* | Coding | I |
| 5383 | T | TC | 2.3 | Insertion | *MT-ND2* | Coding | I |
| 5436 | AC | A | 3.8 | Deletion | *MT-ND2* | Coding | I |
| 5491 | C | CT | 2.7 | Insertion | *MT-ND2* | Coding | I |
| 5507 | CT | C | 2.1 | Deletion | *MT-ND2* | Coding | I |
| 5763 | GC | G | 8 | Deletion | *MT-TC* | tRNA |  |
| 5829 | T | TA | 4.9 | Insertion | *MT-TY* | tRNA |  |
| 6152 | T | TC | 2 | Insertion | *MT-CO1* | Coding | IV |
| 6446 | G | GC | 3 | Insertion | *MT-CO1* | Coding | IV |
| 6572 | CGGA | C | 6.9 | Deletion | *MT-CO1* | Coding | IV |
| 6691 | G | GA | 6 | Insertion | *MT-CO1* | Coding | IV |
| 7091 | AT | A | 13.2 | Deletion | *MT-CO1* | Coding | IV |
| 7180 | TC | T | 10.3 | Deletion | *MT-CO1* | Coding | IV |
| 7215 | CG | C | 3.7 | Deletion | *MT-CO1* | Coding | IV |
| 7231 | AC | A | 4.7 | Deletion | *MT-CO1* | Coding | IV |
| 7341 | C | CG | 5.8 | Insertion | *MT-CO1* | Coding | IV |
| 7396 | G | GC | 2.5 | Insertion | *MT-CO1* | Coding | IV |
| 7446 | C | CA | 2 | Insertion | *MT-TS1* | tRNA |  |
| 7465 | A | AC | 5.2 | Insertion | *MT-TS1* | tRNA |  |
| 7514 | C | CA | 3.5 | Insertion | *MT-TS1* | tRNA |  |
| 7614 | AAG | A | 2.8 | Deletion | *MT-CO2* | Coding | IV |
| 7709 | CT | C | 13.9 | Deletion | *MT-CO2* | Coding | IV |
| 7842 | TA | T | 2.8 | Deletion | *MT-CO2* | Coding | IV |
| 7954 | T | TC | 4.7 | Insertion | *MT-CO2* | Coding | IV |
| 8006 | CG | C | 4.5 | Deletion | *MT-CO2* | Coding | IV |
| 8027 | GC | G | 6.3 | Deletion | *MT-CO2* | Coding | IV |
| 8151 | CG | C | 7.1 | Deletion | *MT-CO2* | Coding | IV |
| 8232 | T | TA | 6.6 | Insertion | *MT-CO2* | Coding | IV |
| 8271 | AC | A | 5.9 | Deletion | NA | - |  |
| 8280 | AC | A | 5.3 | Deletion | NA | - |  |
| 8449 | AT | A | 6.3 | Deletion | *MT-AYP8* | Coding | V |
| 8490 | TA | T | 3.5 | Deletion | *MT-AYP8* | Coding | V |
| 8557 | G | GC | 3.9 | Insertion | *MT-AYP8* | Coding | V |
| 8604 | TC | T | 8.8 | Deletion | *MT-ATP6* | Coding | V |
| 8618 | T | TC | 3.2 | Insertion | *MT-ATP6* | Coding | V |
| 8931 | AC | A | 2.9 | Deletion | *MT-ATP6* | Coding | V |
| 9262 | CA | C | 2.8 | Deletion | *MT-CO3* | Coding | IV |
| 9277 | C | CA | 2.7 | Insertion | *MT-CO3* | Coding | IV |

**S8 Table. INDELs of KKU-100 cell line (cont.)**

| Position | Ref | Variant | Heteroplasmy level (%) | INDEL | Maplocus | Category | OXPHOS  complex |
| --- | --- | --- | --- | --- | --- | --- | --- |
| 9280 | TC | T | 2.7 | Deletion | *MT-CO3* | Coding | IV |
| 9477 | G | GT | 3.9 | Insertion | *MT-CO3* | Coding | IV |
| 9491 | AG | A | 4.8 | Deletion | *MT-CO3* | Coding | IV |
| 9494 | ATT | A | 4.8 | Deletion | *MT-CO3* | Coding | IV |
| 9531 | A | AC | 2.7 | Insertion | *MT-CO3* | Coding | IV |
| 9721 | AT | A | 4.7 | Deletion | *MT-CO3* | Coding | IV |
| 9794 | AT | A | 6.7 | Deletion | *MT-CO3* | Coding | IV |
| 10047 | C | CA | 6 | Insertion | *MT-TG* | tRNA |  |
| 10089 | A | AC | 2.5 | Insertion | *MT-ND3* | Coding | I |
| 10191 | T | TC | 4.2 | Insertion | *MT-ND3* | Coding | I |
| 10357 | T | TA | 5.1 | Insertion | *MT-ND3* | Coding | I |
| 10380 | CA | C | 3.4 | Deletion | *MT-ND3* | Coding | I |
| 10631 | C | CT | 5 | Insertion | *MT-ND4L* | Coding | I |
| 10687 | TG | T | 6.6 | Deletion | *MT-ND4L* | Coding | I |
| 10813 | C | CA | 2 | Insertion | *MT-ND4* | Coding | I |
| 10879 | AT | A | 2.9 | Deletion | *MT-ND4* | Coding | I |
| 10916 | T | TC | 2.9 | Insertion | *MT-ND4* | Coding | I |
| 10935 | AC | A | 15.1 | Deletion | *MT-ND4* | Coding | I |
| 10946 | A | AC | 2.3 | Insertion | *MT-ND4* | Coding | I |
| 11031 | GAA | G | 5 | Deletion | *MT-ND4* | Coding | I |
| 11031 | G | GA | 2.5 | Insertion | *MT-ND4* | Coding | I |
| 11137 | TATC | T | 2.6 | Deletion | *MT-ND4* | Coding | I |
| 11141 | C | CTG | 2.6 | Insertion | *MT-ND4* | Coding | I |
| 11165 | TGA | T | 2.3 | Deletion | *MT-ND4* | Coding | I |
| 11233 | T | TC | 5.1 | Insertion | *MT-ND4* | Coding | I |
| 11280 | TA | T | 5.9 | Deletion | *MT-ND4* | Coding | I |
| 11426 | GC | G | 3.2 | Deletion | *MT-ND4* | Coding | I |
| 11466 | T | TA | 4.9 | Insertion | *MT-ND4* | Coding | I |
| 11604 | TA | T | 2.6 | Deletion | *MT-ND4* | Coding | I |
| 11788 | CCT | C | 4 | Deletion | *MT-ND4* | Coding | I |
| 11865 | TAC | T | 2.2 | Deletion | *MT-ND4* | Coding | I |
| 11866 | A | AC | 2.1 | Insertion | *MT-ND4* | Coding | I |
| 11980 | CCT | C | 2 | Deletion | *MT-ND4* | Coding | I |
| 12083 | TC | T | 33.2 | Deletion | *MT-ND4* | Coding | I |
| 12236 | GC | G | 5.2 | Deletion | *MT-TS2* | tRNA |  |
| 12305 | CA | C | 2.9 | Deletion | *MT-TL2* | tRNA |  |
| 12384 | T | TC | 3.1 | Insertion | *MT-ND5* | Coding | I |
| 12417 | C | CA | 8.7 | Insertion | *MT-ND5* | Coding | I |
| 13124 | T | TC | 3.7 | Insertion | *MT-ND5* | Coding | I |
| 13127 | AC | A | 11.6 | Deletion | *MT-ND5* | Coding | I |
| 13133 | T | TA | 3.6 | Insertion | *MT-ND5* | Coding | I |
| 13230 | CAAA | C | 5.6 | Deletion | *MT-ND5* | Coding | I |
| 13230 | C | CA | 2.3 | Insertion | *MT-ND5* | Coding | I |
| 13365 | CG | C | 2.7 | Deletion | *MT-ND5* | Coding | I |
| 13370 | C | CT | 2.7 | Insertion | *MT-ND5* | Coding | I |
| 13404 | TC | T | 5.2 | Deletion | *MT-ND5* | Coding | I |
| 13406 | G | GA | 2.8 | Insertion | *MT-ND5* | Coding | I |
| 13646 | T | TC | 4.4 | Insertion | *MT-ND5* | Coding | I |
| 13753 | T | TC | 5.2 | Insertion | *MT-ND5* | Coding | I |
| 13793 | T | TA | 3.6 | Insertion | *MT-ND5* | Coding | I |

**S8 Table. INDELs of KKU-100 cell line (cont.)**

| Position | Ref | Variant | Heteroplasmy level (%) | INDEL | Maplocus | Category | OXPHOS  complex |
| --- | --- | --- | --- | --- | --- | --- | --- |
| 13980 | GC | G | 9.8 | Deletion | *MT-ND5* | Coding | I |
| 14074 | CA | C | 3.3 | Deletion | *MT-ND5* | Coding | I |
| 14105 | C | CT | 5.4 | Insertion | *MT-ND5* | Coding | I |
| 14154 | TC | T | 12.5 | Deletion | *MT-ND6* | Coding | I |
| 14243 | GC | G | 2 | Deletion | *MT-ND6* | Coding | I |
| 14264 | T | TC | 2.6 | Insertion | *MT-ND6* | Coding | I |
| 14339 | AC | A | 5.4 | Deletion | *MT-ND6* | Coding | I |
| 14395 | T | TA | 2.2 | Insertion | *MT-ND6* | Coding | I |
| 14488 | TC | T | 2.7 | Deletion | *MT-ND6* | Coding | I |
| 14503 | TA | T | 5.5 | Deletion | *MT-ND6* | Coding | I |
| 14530 | T | TC | 3.9 | Insertion | *MT-ND6* | Coding | I |
| 14808 | T | TC | 4.8 | Insertion | *MT-CYB* | Coding | III |
| 14813 | AC | A | 9.1 | Deletion | *MT-CYB* | Coding | III |
| 15383 | T | TC | 4.1 | Insertion | *MT-CYB* | Coding | III |
| 15541 | T | TC | 4.8 | Insertion | *MT-CYB* | Coding | III |
| 15590 | CG | C | 2.3 | Deletion | *MT-CYB* | Coding | III |
| 15939 | CT | C | 4.5 | Deletion | *MT-TT* | tRNA |  |
| 15961 | G | GA | 4.6 | Insertion | *MT-TP* | tRNA |  |
| 16161 | TA | T | 2.1 | Deletion | MT-DLOOP1 | CR |  |
| 16189 | T | TC | 2.9 | Insertion | MT-DLOOP1 | CR |  |
| 16374 | AC | A | 6.8 | Deletion | MT-DLOOP1 | CR |  |
| 16431 | CA | C | 5 | Deletion | MT-DLOOP1 | CR |  |
| 16469 | TG | T | 6.2 | Deletion | MT-DLOOP1 | CR |  |
| 16535 | GC | G | 4.5 | Deletion | MT-DLOOP1 | CR |  |

**S9 Table. INDELs of KKU-213A cell line**

| Position | Ref | Variant | Heteroplasmy level (%) | INDEL | Maplocus | Category | OXPHOS  complex |
| --- | --- | --- | --- | --- | --- | --- | --- |
| 33 | CG | C | 11.6 | Deletion | MT-DLOOP2 | CR |  |
| 64 | CTG | C | 3.8 | Deletion | MT-DLOOP2 | CR |  |
| 132 | CT | C | 3.8 | Deletion | MT-DLOOP2 | CR |  |
| 247 | GA | G | 96.3 | Deletion | MT-DLOOP2 | CR |  |
| 302 | A | AC | 51.8 | Insertion | MT-DLOOP2 | CR |  |
| 302 | A | ACCC | 8.4 | Insertion | MT-DLOOP2 | CR |  |
| 310 | T | TC | 62.3 | Insertion | MT-DLOOP2 | CR |  |
| 319 | T | TA | 95.4 | Insertion | MT-DLOOP2 | CR |  |
| 356 | C | CA | 2.4 | Insertion | MT-DLOOP2 | CR |  |
| 432 | A | AC | 4 | Insertion | MT-DLOOP2 | CR |  |
| 455 | TC | T | 2.9 | Deletion | MT-DLOOP2 | CR |  |
| 460 | T | TC | 2.5 | Insertion | MT-DLOOP2 | CR |  |
| 493 | A | AC | 4.9 | Insertion | MT-DLOOP2 | CR |  |
| 513 | GCA | G | 3.7 | Deletion | MT-DLOOP2 | CR |  |
| 540 | A | AC | 3.6 | Insertion | MT-DLOOP2 | CR |  |
| 567 | AC | A | 15.6 | Deletion | MT-DLOOP2 | CR |  |
| 949 | TA | T | 4.7 | Deletion | *MT-RNR1* | rRNA |  |
| 955 | AC | A | 9.2 | Deletion | *MT-RNR1* | rRNA |  |
| 961 | T | TC | 7.1 | Insertion | *MT-RNR1* | rRNA |  |
| 992 | T | TA | 3.6 | Insertion | *MT-RNR1* | rRNA |  |
| 1165 | C | CA | 3.4 | Insertion | *MT-RNR1* | rRNA |  |
| 1374 | A | AC | 3.3 | Insertion | *MT-RNR1* | rRNA |  |
| 1547 | TA | T | 3.3 | Deletion | *MT-RNR1* | rRNA |  |
| 1597 | CG | C | 5.2 | Deletion | *MT-RNR1* | rRNA |  |
| 1623 | G | GA | 15.6 | Insertion | *MT-TV* | tRNA |  |
| 1627 | C | CGA | 15.6 | Insertion | *MT-TV* | tRNA |  |
| 1633 | T | TTCTTC | 13.2 | Insertion | *MT-TV* | tRNA |  |
| 1636 | A | ACCT | 12.3 | Insertion | *MT-TV* | tRNA |  |
| 1790 | A | AG | 2.1 | Insertion | *MT-RNR2* | rRNA |  |
| 1800 | GA | G | 3.8 | Deletion | *MT-RNR2* | rRNA |  |
| 1900 | AC | A | 21.5 | Deletion | *MT-RNR2* | rRNA |  |
| 1934 | TA | T | 7.6 | Deletion | *MT-RNR2* | rRNA |  |
| 2101 | C | CA | 4.6 | Insertion | *MT-RNR2* | rRNA |  |
| 2129 | GA | G | 2.8 | Deletion | *MT-RNR2* | rRNA |  |
| 2177 | T | TA | 2.6 | Insertion | *MT-RNR2* | rRNA |  |
| 2226 | T | TA | 87.9 | Insertion | *MT-RNR2* | rRNA |  |
| 2254 | CCT | C | 4.1 | Deletion | *MT-RNR2* | rRNA |  |
| 2347 | CA | C | 3.6 | Deletion | *MT-RNR2* | rRNA |  |
| 2456 | T | TA | 3.7 | Insertion | *MT-RNR2* | rRNA |  |
| 2465 | TA | T | 4.1 | Deletion | *MT-RNR2* | rRNA |  |
| 2487 | AC | A | 13.2 | Deletion | *MT-RNR2* | rRNA |  |
| 2502 | C | CA | 2.4 | Insertion | *MT-RNR2* | rRNA |  |
| 2686 | G | GC | 2.7 | Insertion | *MT-RNR2* | rRNA |  |
| 2813 | TG | T | 6 | Deletion | *MT-RNR2* | rRNA |  |
| 3027 | TA | T | 4.9 | Deletion | *MT-RNR2* | rRNA |  |
| 3126 | CG | C | 3.1 | Deletion | *MT-RNR2* | rRNA |  |
| 3167 | TC | T | 9.4 | Deletion | *MT-RNR2* | rRNA |  |
| 3244 | G | GC | 3.1 | Insertion | *MT-TL1* | tRNA |  |
| 3380 | GA | G | 4.9 | Deletion | *MT-ND1* | Coding | I |
| 3565 | AC | A | 5.9 | Deletion | *MT-ND1* | Coding | I |

**S9 Table. INDELs of KKU-213A cell line (cont.)**

| Position | Ref | Variant | Heteroplasmy level (%) | INDEL | Maplocus | Category | OXPHOS  complex |
| --- | --- | --- | --- | --- | --- | --- | --- |
| 3572 | T | TC | 8.9 | Insertion | *MT-ND1* | Coding | I |
| 3584 | AC | A | 5.6 | Deletion | *MT-ND1* | Coding | I |
| 4057 | TC | T | 5.6 | Deletion | *MT-ND1* | Coding | I |
| 4136 | A | AC | 2.3 | Insertion | *MT-ND1* | Coding | I |
| 4248 | TC | T | 6.5 | Deletion | *MT-ND1* | Coding | I |
| 4425 | TC | T | 2.7 | Deletion | *MT-TM* | tRNA |  |
| 4435 | AC | A | 3.9 | Deletion | *MT-TM* | tRNA |  |
| 4460 | T | TC | 5.9 | Insertion | *MT-TM* | tRNA |  |
| 4475 | TC | T | 3.7 | Deletion | *MT-ND2* | Coding | I |
| 4604 | C | CA | 3.8 | Insertion | *MT-ND2* | Coding | I |
| 4714 | GA | G | 7.8 | Deletion | *MT-ND2* | Coding | I |
| 4780 | TA | T | 2.3 | Deletion | *MT-ND2* | Coding | I |
| 4794 | GC | G | 7.9 | Deletion | *MT-ND2* | Coding | I |
| 4869 | CA | C | 3.6 | Deletion | *MT-ND2* | Coding | I |
| 4878 | G | GC | 2.6 | Insertion | *MT-ND2* | Coding | I |
| 5217 | T | TC | 6.9 | Insertion | *MT-ND2* | Coding | I |
| 5231 | GC | G | 9.8 | Deletion | *MT-ND2* | Coding | I |
| 5281 | C | CA | 5.7 | Insertion | *MT-ND2* | Coding | I |
| 5446 | TC | T | 3.5 | Deletion | *MT-ND2* | Coding | I |
| 5487 | T | TC | 2 | Insertion | *MT-ND2* | Coding | I |
| 5490 | CCT | C | 3.8 | Deletion | *MT-ND2* | Coding | I |
| 5743 | CG | C | 20.3 | Deletion | NA | - |  |
| 5746 | GA | G | 5.7 | Deletion | NA | - |  |
| 5763 | GC | G | 12.7 | Deletion | *MT-TC* | tRNA |  |
| 5829 | TA | T | 9.3 | Deletion | *MT-TY* | tRNA |  |
| 5851 | C | CT | 4.4 | Insertion | *MT-TY* | tRNA |  |
| 5884 | AT | A | 5.4 | Deletion | *MT-TY* | tRNA |  |
| 5968 | TC | T | 3.8 | Deletion | *MT-CO1* | Coding | IV |
| 6152 | TC | T | 10.3 | Deletion | *MT-CO1* | Coding | IV |
| 6221 | TC | T | 3 | Deletion | *MT-CO1* | Coding | IV |
| 6223 | CCT | C | 4.5 | Deletion | *MT-CO1* | Coding | IV |
| 6271 | GA | G | 8.5 | Deletion | *MT-CO1* | Coding | IV |
| 6499 | T | TC | 5.4 | Insertion | *MT-CO1* | Coding | IV |
| 6565 | AC | A | 5.3 | Deletion | *MT-CO1* | Coding | IV |
| 6583 | A | AC | 8.2 | Insertion | *MT-CO1* | Coding | IV |
| 6691 | G | GA | 8.9 | Insertion | *MT-CO1* | Coding | IV |
| 6942 | CT | C | 12.2 | Deletion | *MT-CO1* | Coding | IV |
| 7091 | A | AT | 3.1 | Insertion | *MT-CO1* | Coding | IV |
| 7094 | TC | T | 9.3 | Deletion | *MT-CO1* | Coding | IV |
| 7180 | TC | T | 14.4 | Deletion | *MT-CO1* | Coding | IV |
| 7211 | GC | G | 9.4 | Deletion | *MT-CO1* | Coding | IV |
| 7231 | AC | A | 6.2 | Deletion | *MT-CO1* | Coding | IV |
| 7341 | C | CG | 9.2 | Insertion | *MT-CO1* | Coding | IV |
| 7396 | GC | G | 20.4 | Deletion | *MT-CO1* | Coding | IV |
| 7446 | CA | C | 8.7 | Deletion | *MT-TS1* | tRNA |  |
| 7465 | A | AC | 5.5 | Insertion | *MT-TS1* | tRNA |  |
| 7514 | CA | C | 13.1 | Deletion | *MT-TS1* | tRNA |  |
| 7625 | TC | T | 6.7 | Deletion | *MT-CO2* | Coding | IV |
| 7709 | CT | C | 18 | Deletion | *MT-CO2* | Coding | IV |
| 7842 | TA | T | 5 | Deletion | *MT-CO2* | Coding | IV |

**S9 Table. INDELs of KKU-213A cell line (cont.)**

| Position | Ref | Variant | Heteroplasmy level (%) | INDEL | Maplocus | Category | OXPHOS  complex |
| --- | --- | --- | --- | --- | --- | --- | --- |
| 7954 | TC | T | 10.5 | Deletion | *MT-CO2* | Coding | IV |
| 8079 | TC | T | 15.5 | Deletion | *MT-CO2* | Coding | IV |
| 8151 | CG | C | 7.5 | Deletion | *MT-CO2* | Coding | IV |
| 8232 | TA | T | 5.8 | Deletion | *MT-CO2* | Coding | IV |
| 8280 | AC | A | 8.7 | Deletion | NA | - |  |
| 8341 | AG | A | 3.4 | Deletion | *MT-TK* | tRNA |  |
| 8490 | T | TA | 3.8 | Insertion | *MT-ATP8* | Coding | V |
| 8604 | TC | T | 17.6 | Deletion | *MT-ATP6* | Coding | V |
| 8931 | AC | A | 2.6 | Deletion | *MT-ATP6* | Coding | V |
| 9291 | T | TC | 4.2 | Insertion | *MT-CO3* | Coding | IV |
| 9449 | CG | C | 7.4 | Deletion | *MT-CO3* | Coding | IV |
| 9477 | GT | G | 3.3 | Deletion | *MT-CO3* | Coding | IV |
| 9501 | T | TG | 2.8 | Insertion | *MT-CO3* | Coding | IV |
| 9531 | A | AC | 3.7 | Insertion | *MT-CO3* | Coding | IV |
| 9544 | GA | G | 4.3 | Deletion | *MT-CO3* | Coding | IV |
| 9663 | GA | G | 5.1 | Deletion | *MT-CO3* | Coding | IV |
| 9721 | AT | A | 5.9 | Deletion | *MT-CO3* | Coding | IV |
| 9794 | A | AT | 2.4 | Insertion | *MT-CO3* | Coding | IV |
| 9994 | CT | C | 3.1 | Deletion | *MT-TG* | tRNA |  |
| 10047 | C | CA | 7.4 | Insertion | *MT-TG* | tRNA |  |
| 10191 | TC | T | 5.5 | Deletion | *MT-ND3* | Coding | I |
| 10197 | G | GCC | 2.9 | Insertion | *MT-ND3* | Coding | I |
| 10204 | T | TC | 4.3 | Insertion | *MT-ND3* | Coding | I |
| 10244 | C | CT | 2.2 | Insertion | *MT-ND3* | Coding | I |
| 10272 | CT | C | 10 | Deletion | *MT-ND3* | Coding | I |
| 10357 | T | TA | 5.4 | Insertion | *MT-ND3* | Coding | I |
| 10380 | CA | C | 2.2 | Deletion | *MT-ND3* | Coding | I |
| 10879 | AT | A | 4.7 | Deletion | *MT-ND4* | Coding | I |
| 10916 | T | TC | 3 | Insertion | *MT-ND4* | Coding | I |
| 10935 | AC | A | 13.6 | Deletion | *MT-ND4* | Coding | I |
| 10946 | A | AC | 3.3 | Insertion | *MT-ND4* | Coding | I |
| 10978 | AC | A | 3.3 | Deletion | *MT-ND4* | Coding | I |
| 11031 | GA | G | 10.9 | Deletion | *MT-ND4* | Coding | I |
| 11233 | T | TC | 3.7 | Insertion | *MT-ND4* | Coding | I |
| 11280 | TA | T | 5.3 | Deletion | *MT-ND4* | Coding | I |
| 11306 | G | GC | 2.5 | Insertion | *MT-ND4* | Coding | I |
| 11437 | T | TG | 2.6 | Insertion | *MT-ND4* | Coding | I |
| 11466 | T | TA | 3.3 | Insertion | *MT-ND4* | Coding | I |
| 11511 | AC | A | 5.3 | Deletion | *MT-ND4* | Coding | I |
| 11604 | TA | T | 2.4 | Deletion | *MT-ND4* | Coding | I |
| 11621 | TA | T | 5.6 | Deletion | *MT-ND4* | Coding | I |
| 11625 | CT | C | 4.8 | Deletion | *MT-ND4* | Coding | I |
| 11788 | CCT | C | 4.1 | Deletion | *MT-ND4* | Coding | I |
| 11866 | A | AC | 3.1 | Insertion | *MT-ND4* | Coding | I |
| 12005 | TG | T | 3.2 | Deletion | *MT-ND4* | Coding | I |
| 12083 | TC | T | 30.5 | Deletion | *MT-ND4* | Coding | I |
| 12135 | CT | C | 5.4 | Deletion | *MT-ND4* | Coding | I |
| 12200 | A | AT | 2.1 | Insertion | *MT-TH* | tRNA |  |
| 12206 | CGAG | C | 3.6 | Deletion | *MT-TH* | tRNA |  |
| 12236 | GC | G | 6.6 | Deletion | *MT-TS2* | tRNA |  |

**S9 Table. INDELs of KKU-213A cell line (cont.)**

| Position | Ref | Variant | Heteroplasmy level (%) | INDEL | Maplocus | Category | OXPHOS  complex |
| --- | --- | --- | --- | --- | --- | --- | --- |
| 12305 | CA | C | 2.5 | Deletion | *MT-TL2* | tRNA |  |
| 12384 | T | TC | 2.7 | Insertion | *MT-ND5* | Coding | I |
| 12417 | C | CA | 7.5 | Insertion | *MT-ND5* | Coding | I |
| 12565 | T | TC | 2.6 | Insertion | *MT-ND5* | Coding | I |
| 13025 | AC | A | 3.1 | Deletion | *MT-ND5* | Coding | I |
| 13127 | AC | A | 9.8 | Deletion | *MT-ND5* | Coding | I |
| 13230 | CA | C | 3.5 | Deletion | *MT-ND5* | Coding | I |
| 13365 | CG | C | 3.5 | Deletion | *MT-ND5* | Coding | I |
| 13370 | C | CT | 4.6 | Insertion | *MT-ND5* | Coding | I |
| 13406 | G | GA | 3.4 | Insertion | *MT-ND5* | Coding | I |
| 13646 | T | TC | 5.3 | Insertion | *MT-ND5* | Coding | I |
| 13753 | T | TC | 4.5 | Insertion | *MT-ND5* | Coding | I |
| 13820 | T | TC | 10.9 | Insertion | *MT-ND5* | Coding | I |
| 13842 | AG | A | 2.9 | Deletion | *MT-ND5* | Coding | I |
| 13980 | GC | G | 12.6 | Deletion | *MT-ND5* | Coding | I |
| 14105 | C | CT | 7.3 | Insertion | *MT-ND5* | Coding | I |
| 14114 | T | TC | 3.3 | Insertion | *MT-ND5* | Coding | I |
| 14154 | TC | T | 18.4 | Deletion | *MT-ND6* | Coding | I |
| 14264 | TC | T | 4.7 | Deletion | *MT-ND6* | Coding | I |
| 14339 | AC | A | 4.2 | Deletion | *MT-ND6* | Coding | I |
| 14395 | T | TA | 3.3 | Insertion | *MT-ND6* | Coding | I |
| 14488 | TC | T | 6.2 | Deletion | *MT-ND6* | Coding | I |
| 14503 | TA | T | 4.9 | Deletion | *MT-ND6* | Coding | I |
| 14530 | T | TC | 5.3 | Insertion | *MT-ND6* | Coding | I |
| 14556 | A | AC | 2.5 | Insertion | *MT-ND6* | Coding | I |
| 14769 | AC | A | 3.1 | Deletion | *MT-CYB* | Coding | III |
| 14808 | T | TC | 4.3 | Insertion | *MT-CYB* | Coding | III |
| 14813 | AC | A | 11.5 | Deletion | *MT-CYB* | Coding | III |
| 15204 | TC | T | 3.6 | Deletion | *MT-CYB* | Coding | III |
| 15221 | GA | G | 2.4 | Deletion | *MT-CYB* | Coding | III |
| 15224 | C | CT | 2.8 | Insertion | *MT-CYB* | Coding | III |
| 15349 | CG | C | 6.2 | Deletion | *MT-CYB* | Coding | III |
| 15383 | T | TC | 2.5 | Insertion | *MT-CYB* | Coding | III |
| 15536 | A | AC | 20.6 | Insertion | *MT-CYB* | Coding | III |
| 15536 | A | ACCC | 7.7 | Insertion | *MT-CYB* | Coding | III |
| 15536 | A | ACCCCC | 4.5 | Insertion | *MT-CYB* | Coding | III |
| 15540 | CT | C | 19 | Deletion | *MT-CYB* | Coding | III |
| 15590 | CG | C | 5.6 | Deletion | *MT-CYB* | Coding | III |
| 15961 | G | GA | 4.6 | Insertion | *MT-TP* | tRNA |  |
| 16179 | CA | C | 20.1 | Deletion | MT-DLOOP1 | CR |  |
| 16182 | A | AC | 16.2 | Insertion | MT-DLOOP1 | CR |  |
| 16182 | A | ACCC | 8.6 | Insertion | MT-DLOOP1 | CR |  |
| 16182 | A | ACCCCC | 3.5 | Insertion | MT-DLOOP1 | CR |  |
| 16187 | CCT | C | 3 | Deletion | MT-DLOOP1 | CR |  |
| 16289 | A | AC | 2 | Insertion | MT-DLOOP1 | CR |  |
| 16374 | AC | A | 4.4 | Deletion | MT-DLOOP1 | CR |  |
| 16431 | CA | C | 4.9 | Deletion | MT-DLOOP1 | CR |  |
| 16469 | TG | T | 7 | Deletion | MT-DLOOP1 | CR |  |

**S10 Table. INDELs of KKU-452 cell line.**

| Position | Ref | Variant | Heteroplasmy level (%) | INDEL | Maplocus | Category | OXPHOS  complex |
| --- | --- | --- | --- | --- | --- | --- | --- |
| 33 | CG | C | 9.3 | Deletion | MT-DLOOP2 | CR |  |
| 49 | AT | A | 5.7 | Deletion | MT-DLOOP2 | CR |  |
| 65 | TGG | T | 2.6 | Deletion | MT-DLOOP2 | CR |  |
| 65 | T | TG | 2.3 | Insertion | MT-DLOOP2 | CR |  |
| 71 | GTA | G | 3.2 | Deletion | MT-DLOOP2 | CR |  |
| 302 | A | AC | 63.7 | Insertion | MT-DLOOP2 | CR |  |
| 302 | A | ACCC | 8.6 | Insertion | MT-DLOOP2 | CR |  |
| 310 | T | TC | 77.4 | Insertion | MT-DLOOP2 | CR |  |
| 432 | A | AC | 5 | Insertion | MT-DLOOP2 | CR |  |
| 493 | A | AC | 3.1 | Insertion | MT-DLOOP2 | CR |  |
| 499 | G | GCC | 2.6 | Insertion | MT-DLOOP2 | CR |  |
| 513 | GCA | G | 2.6 | Deletion | MT-DLOOP2 | CR |  |
| 540 | A | AC | 2.4 | Insertion | MT-DLOOP2 | CR |  |
| 560 | C | CA | 2.7 | Insertion | MT-DLOOP2 | CR |  |
| 567 | AC | A | 8.6 | Deletion | MT-DLOOP2 | CR |  |
| 636 | AC | A | 2.4 | Deletion | *MT-TF* | tRNA |  |
| 808 | CG | C | 5.5 | Deletion | *MT-RNR1* | rRNA |  |
| 955 | A | AC | 19.2 | Insertion | *MT-RNR1* | rRNA |  |
| 955 | A | ACCC | 5.8 | Insertion | *MT-RNR1* | rRNA |  |
| 959 | CCT | C | 10.2 | Deletion | *MT-RNR1* | rRNA |  |
| 992 | T | TA | 4.6 | Insertion | *MT-RNR1* | rRNA |  |
| 1374 | A | AC | 3.8 | Insertion | *MT-RNR1* | rRNA |  |
| 1547 | TA | T | 4.5 | Deletion | *MT-RNR1* | rRNA |  |
| 1597 | CG | C | 10.4 | Deletion | *MT-RNR1* | rRNA |  |
| 1800 | GA | G | 3.3 | Deletion | *MT-RNR2* | rRNA |  |
| 1899 | GAC | G | 3.6 | Deletion | *MT-RNR2* | rRNA |  |
| 1900 | AC | A | 17.1 | Deletion | *MT-RNR2* | rRNA |  |
| 1934 | T | TA | 2.2 | Insertion | *MT-RNR2* | rRNA |  |
| 2101 | C | CA | 6 | Insertion | *MT-RNR2* | rRNA |  |
| 2129 | GA | G | 5.9 | Deletion | *MT-RNR2* | rRNA |  |
| 2177 | T | TA | 6.1 | Insertion | *MT-RNR2* | rRNA |  |
| 2226 | T | TA | 2.3 | Insertion | *MT-RNR2* | rRNA |  |
| 2254 | CCT | C | 4.4 | Deletion | *MT-RNR2* | rRNA |  |
| 2347 | CA | C | 6 | Deletion | *MT-RNR2* | rRNA |  |
| 2352 | T | TA | 2 | Insertion | *MT-RNR2* | rRNA |  |
| 2456 | T | TA | 3.5 | Insertion | *MT-RNR2* | rRNA |  |
| 2487 | AC | A | 10.1 | Deletion | *MT-RNR2* | rRNA |  |
| 2496 | G | GT | 3.9 | Insertion | *MT-RNR2* | rRNA |  |
| 2800 | TA | T | 2.5 | Deletion | *MT-RNR2* | rRNA |  |
| 3126 | CG | C | 2.9 | Deletion | *MT-RNR2* | rRNA |  |
| 3167 | TC | T | 9.6 | Deletion | *MT-RNR2* | rRNA |  |
| 3483 | G | GC | 2.5 | Insertion | *MT-ND1* | Coding | I |
| 3572 | T | TC | 4.7 | Insertion | *MT-ND1* | Coding | I |
| 3584 | AC | A | 4.9 | Deletion | *MT-ND1* | Coding | I |
| 3783 | CT | C | 6.8 | Deletion | *MT-ND1* | Coding | I |
| 4136 | A | AC | 2.2 | Insertion | *MT-ND1* | Coding | I |
| 4175 | G | GA | 2.6 | Insertion | *MT-ND1* | Coding | I |
| 4248 | TC | T | 4.5 | Deletion | *MT-ND1* | Coding | I |
| 4794 | GC | G | 5.3 | Deletion | *MT-ND2* | Coding | I |
| 4869 | CA | C | 6 | Deletion | *MT-ND2* | Coding | I |

**S10 Table. INDELs of KKU-452 cell line (cont.).**

| Position | Ref | Variant | Heteroplasmy level (%) | INDEL | Maplocus | Category | OXPHOS  complex |
| --- | --- | --- | --- | --- | --- | --- | --- |
| 4878 | GC | G | 14.8 | Deletion | *MT-ND2* | Coding | I |
| 4939 | TC | T | 2.1 | Deletion | *MT-ND2* | Coding | I |
| 5220 | C | CT | 4.9 | Insertion | *MT-ND2* | Coding | I |
| 5231 | G | GC | 2.9 | Insertion | *MT-ND2* | Coding | I |
| 5281 | CA | C | 2 | Deletion | *MT-ND2* | Coding | I |
| 5491 | C | CT | 2.1 | Insertion | *MT-ND2* | Coding | I |
| 5507 | CT | C | 2.6 | Deletion | *MT-ND2* | Coding | I |
| 5743 | CG | C | 17.7 | Deletion | NA | - |  |
| 5763 | GC | G | 12.1 | Deletion | *MT-TC* | tRNA |  |
| 5829 | T | TA | 5 | Insertion | *MT-TY* | tRNA |  |
| 5986 | TC | T | 6.9 | Deletion | *MT-CO1* | Coding | IV |
| 6152 | T | TC | 3 | Insertion | *MT-CO1* | Coding | IV |
| 6221 | T | TC | 3.1 | Insertion | *MT-CO1* | Coding | IV |
| 6223 | C | CCT | 2.2 | Insertion | *MT-CO1* | Coding | IV |
| 6446 | G | GC | 2.8 | Insertion | *MT-CO1* | Coding | IV |
| 6583 | A | AC | 5.2 | Insertion | *MT-CO1* | Coding | IV |
| 6691 | G | GA | 6.9 | Insertion | *MT-CO1* | Coding | IV |
| 7094 | TC | T | 7.9 | Deletion | *MT-CO1* | Coding | IV |
| 7180 | TC | T | 11.9 | Deletion | *MT-CO1* | Coding | IV |
| 7231 | AC | A | 6.8 | Deletion | *MT-CO1* | Coding | IV |
| 7341 | C | CG | 10.5 | Insertion | *MT-CO1* | Coding | IV |
| 7396 | G | GC | 2.5 | Insertion | *MT-CO1* | Coding | IV |
| 7446 | C | CA | 3.2 | Insertion | *MT-TS1* | tRNA |  |
| 7465 | A | AC | 2.2 | Insertion | *MT-TS1* | tRNA |  |
| 7508 | C | CT | 2.6 | Insertion | *MT-TS1* | tRNA |  |
| 7514 | C | CA | 4 | Insertion | *MT-TS1* | tRNA |  |
| 7709 | C | CT | 2.6 | Insertion | *MT-CO2* | Coding | IV |
| 7842 | TA | T | 5 | Deletion | *MT-CO2* | Coding | IV |
| 7954 | T | TC | 3.7 | Insertion | *MT-CO2* | Coding | IV |
| 8027 | GC | G | 4.5 | Deletion | *MT-CO2* | Coding | IV |
| 8151 | CG | C | 9.4 | Deletion | *MT-CO2* | Coding | IV |
| 8232 | TA | T | 5 | Deletion | *MT-CO2* | Coding | IV |
| 8271 | AC | A | 4.9 | Deletion | NA | - |  |
| 8280 | AC | A | 9.3 | Deletion | NA | - |  |
| 8354 | CT | C | 9.6 | Deletion | *MT-TK* | tRNA |  |
| 8368 | G | GC | 3 | Insertion | *MT-AYP8* | Coding | V |
| 8449 | AT | A | 2.4 | Deletion | *MT-AYP8* | Coding | V |
| 8490 | T | TA | 3.4 | Insertion | *MT-AYP8* | Coding | V |
| 8557 | G | GC | 3.6 | Insertion | *MT-AYP8* | Coding | V |
| 8604 | TC | T | 9.6 | Deletion | *MT-ATP6* | Coding | V |
| 9291 | T | TC | 4.1 | Insertion | *MT-CO3* | Coding | IV |
| 9477 | G | GT | 2.4 | Insertion | *MT-CO3* | Coding | IV |
| 9531 | A | AC | 2.3 | Insertion | *MT-CO3* | Coding | IV |
| 9721 | AT | A | 4.9 | Deletion | *MT-CO3* | Coding | IV |
| 9794 | AT | A | 6.8 | Deletion | *MT-CO3* | Coding | IV |
| 9994 | C | CT | 2.8 | Insertion | *MT-TG* | tRNA |  |
| 10047 | C | CA | 6.6 | Insertion | *MT-TG* | tRNA |  |
| 10089 | A | AC | 2.1 | Insertion | *MT-ND3* | Coding | I |
| 10191 | TC | T | 3.8 | Deletion | *MT-ND3* | Coding | I |
| 10197 | G | GCC | 2.6 | Insertion | *MT-ND3* | Coding | I |

**S10 Table. INDELs of KKU-452 cell line (cont.).**

| Position | Ref | Variant | Heteroplasmy level (%) | INDEL | Maplocus | Category | OXPHOS  complex |
| --- | --- | --- | --- | --- | --- | --- | --- |
| 10357 | T | TA | 5 | Insertion | *MT-ND3* | Coding | I |
| 10380 | CA | C | 2.5 | Deletion | *MT-ND3* | Coding | I |
| 10394 | C | CT | 2.2 | Insertion | *MT-ND3* | Coding | I |
| 10631 | C | CT | 6 | Insertion | *MT-ND4L* | Coding | I |
| 10687 | TG | T | 7.9 | Deletion | *MT-ND4L* | Coding | I |
| 10879 | AT | A | 2.5 | Deletion | *MT-ND4* | Coding | I |
| 10916 | T | TC | 2.3 | Insertion | *MT-ND4* | Coding | I |
| 10935 | AC | A | 10.6 | Deletion | *MT-ND4* | Coding | I |
| 10946 | A | AC | 5.9 | Insertion | *MT-ND4* | Coding | I |
| 10978 | AC | A | 2 | Deletion | *MT-ND4* | Coding | I |
| 11031 | GAA | G | 4.8 | Deletion | *MT-ND4* | Coding | I |
| 11031 | G | GA | 2.1 | Insertion | *MT-ND4* | Coding | I |
| 11141 | C | CTG | 2.6 | Insertion | *MT-ND4* | Coding | I |
| 11233 | T | TC | 5.8 | Insertion | *MT-ND4* | Coding | I |
| 11280 | TA | T | 3.1 | Deletion | *MT-ND4* | Coding | I |
| 11426 | GC | G | 2 | Deletion | *MT-ND4* | Coding | I |
| 11466 | T | TA | 4.3 | Insertion | *MT-ND4* | Coding | I |
| 11625 | CT | C | 4.3 | Deletion | *MT-ND4* | Coding | I |
| 11788 | CCT | C | 4.9 | Deletion | *MT-ND4* | Coding | I |
| 11826 | CT | C | 2.2 | Deletion | *MT-ND4* | Coding | I |
| 11866 | AC | A | 6.9 | Deletion | *MT-ND4* | Coding | I |
| 12083 | TC | T | 32.6 | Deletion | *MT-ND4* | Coding | I |
| 12135 | CT | C | 4.1 | Deletion | *MT-ND4* | Coding | I |
| 12236 | G | GC | 4 | Insertion | *MT-TS2* | tRNA | I |
| 12305 | CA | C | 4.9 | Deletion | *MT-TL2* | tRNA | I |
| 12384 | TC | T | 15.3 | Deletion | *MT-ND5* | Coding | I |
| 12417 | C | CA | 8.2 | Insertion | *MT-ND5* | Coding | I |
| 13057 | A | AC | 3.5 | Insertion | *MT-ND5* | Coding | I |
| 13127 | AC | A | 7.9 | Deletion | *MT-ND5* | Coding | I |
| 13173 | CT | C | 2.6 | Deletion | *MT-ND5* | Coding | I |
| 13230 | C | CA | 2.7 | Insertion | *MT-ND5* | Coding | I |
| 13365 | CG | C | 7.3 | Deletion | *MT-ND5* | Coding | I |
| 13370 | C | CT | 4.3 | Insertion | *MT-ND5* | Coding | I |
| 13405 | CG | C | 3 | Deletion | *MT-ND5* | Coding | I |
| 13406 | GA | G | 4.1 | Deletion | *MT-ND5* | Coding | I |
| 13628 | TA | T | 3.7 | Deletion | *MT-ND5* | Coding | I |
| 13646 | T | TC | 5.6 | Insertion | *MT-ND5* | Coding | I |
| 13753 | T | TC | 3.9 | Insertion | *MT-ND5* | Coding | I |
| 13767 | C | CT | 4.2 | Insertion | *MT-ND5* | Coding | I |
| 14074 | CA | C | 2.8 | Deletion | *MT-ND5* | Coding | I |
| 14105 | C | CT | 5.3 | Insertion | *MT-ND5* | Coding | I |
| 14114 | TC | T | 5.9 | Deletion | *MT-ND5* | Coding | I |
| 14154 | T | TC | 3.1 | Insertion | *MT-ND6* | Coding | I |
| 14264 | TC | T | 19.7 | Deletion | *MT-ND6* | Coding | I |
| 14488 | TC | T | 3.9 | Deletion | *MT-ND6* | Coding | I |
| 14503 | T | TA | 2.6 | Insertion | *MT-ND6* | Coding | I |
| 14530 | T | TC | 5.5 | Insertion | *MT-ND6* | Coding | I |
| 14808 | T | TC | 4.4 | Insertion | *MT-CYB* | Coding | III |
| 15262 | T | TC | 2.8 | Insertion | *MT-CYB* | Coding | III |
| 15366 | ACC | A | 2.3 | Deletion | *MT-CYB* | Coding | III |

**S10 Table. INDELs of KKU-452 cell line (cont.).**

| Position | Ref | Variant | Heteroplasmy level (%) | INDEL | Maplocus | Category | OXPHOS  complex |
| --- | --- | --- | --- | --- | --- | --- | --- |
| 15541 | T | TC | 3 | Insertion | *MT-CYB* | Coding | III |
| 15854 | TC | T | 3.6 | Deletion | *MT-CYB* | Coding | III |
| 15939 | CT | C | 2.2 | Deletion | *MT-TT* | tRNA |  |
| 15961 | G | GA | 4.6 | Insertion | *MT-TP* | tRNA |  |
| 15997 | T | TA | 3.6 | Insertion | *MT-TP* | tRNA |  |
| 16189 | T | TC | 3.9 | Insertion | MT-DLOOP1 | CR |  |
| 16374 | AC | A | 5.8 | Deletion | MT-DLOOP1 | CR |  |
| 16431 | CA | C | 5.5 | Deletion | MT-DLOOP1 | CR |  |
| 16445 | T | TC | 2.2 | Insertion | MT-DLOOP1 | CR |  |
| 16469 | TG | T | 7.3 | Deletion | MT-DLOOP1 | CR |  |

**S11 Table. INDELs of MMNK-1 cell line.**

| Position | Ref | Variant | Heteroplasmy level (%) | INDEL | Maplocus | Category | OXPHOS  complex |
| --- | --- | --- | --- | --- | --- | --- | --- |
| 33 | CG | C | 10 | Deletion | MT-DLOOP2 | CR |  |
| 49 | AT | A | 2.9 | Deletion | MT-DLOOP2 | CR |  |
| 65 | T | TG | 4.6 | Insertion | MT-DLOOP2 | CR |  |
| 71 | GTA | G | 8.3 | Deletion | MT-DLOOP2 | CR |  |
| 145 | CT | C | 8.5 | Deletion | MT-DLOOP2 | CR |  |
| 302 | A | AC | 6.2 | Insertion | MT-DLOOP2 | CR |  |
| 310 | T | TC | 73.4 | Insertion | MT-DLOOP2 | CR |  |
| 432 | AC | A | 4.8 | Deletion | MT-DLOOP2 | CR |  |
| 455 | TC | T | 3.3 | Deletion | MT-DLOOP2 | CR |  |
| 460 | TC | T | 4.5 | Deletion | MT-DLOOP2 | CR |  |
| 493 | A | AC | 3.2 | Insertion | MT-DLOOP2 | CR |  |
| 499 | G | GC | 3.3 | Insertion | MT-DLOOP2 | CR |  |
| 513 | GCA | G | 99.2 | Deletion | MT-DLOOP2 | CR |  |
| 540 | A | AC | 3.9 | Insertion | MT-DLOOP2 | CR |  |
| 560 | CA | C | 12 | Deletion | MT-DLOOP2 | CR |  |
| 567 | A | AC | 3 | Insertion | MT-DLOOP2 | CR |  |
| 597 | C | CA | 2.8 | Insertion | *MT-TF* | tRNA |  |
| 704 | TC | T | 2 | Deletion | *MT-RNR1* | rRNA |  |
| 723 | AC | A | 3.5 | Deletion | *MT-RNR1* | rRNA |  |
| 749 | GA | G | 3.2 | Deletion | *MT-RNR1* | rRNA |  |
| 801 | A | AC | 3.7 | Insertion | *MT-RNR1* | rRNA |  |
| 808 | CG | C | 7.6 | Deletion | *MT-RNR1* | rRNA |  |
| 955 | AC | A | 5.2 | Deletion | *MT-RNR1* | rRNA |  |
| 961 | T | TC | 5.8 | Insertion | *MT-RNR1* | rRNA |  |
| 992 | T | TA | 4.2 | Insertion | *MT-RNR1* | rRNA |  |
| 1374 | A | AC | 5.5 | Insertion | *MT-RNR1* | rRNA |  |
| 1547 | TA | T | 4.8 | Deletion | *MT-RNR1* | rRNA |  |
| 1597 | CG | C | 8 | Deletion | *MT-RNR1* | rRNA |  |
| 1800 | GA | G | 3.8 | Deletion | *MT-RNR2* | rRNA |  |
| 1900 | AC | A | 20 | Deletion | *MT-RNR2* | rRNA |  |
| 1934 | T | TA | 2.2 | Insertion | *MT-RNR2* | rRNA |  |
| 2129 | GA | G | 3.8 | Deletion | *MT-RNR2* | rRNA |  |
| 2177 | T | TA | 5.4 | Insertion | *MT-RNR2* | rRNA |  |
| 2254 | CCT | C | 4.4 | Deletion | *MT-RNR2* | rRNA |  |
| 2347 | CA | C | 6 | Deletion | *MT-RNR2* | rRNA |  |
| 2456 | T | TA | 6.2 | Insertion | *MT-RNR2* | rRNA |  |
| 2465 | TA | T | 4.6 | Deletion | *MT-RNR2* | rRNA |  |
| 2487 | AC | A | 13.1 | Deletion | *MT-RNR2* | rRNA |  |
| 2496 | G | GT | 2.4 | Insertion | *MT-RNR2* | rRNA |  |
| 2813 | TG | T | 6.9 | Deletion | *MT-RNR2* | rRNA |  |
| 3027 | TA | T | 2.9 | Deletion | *MT-RNR2* | rRNA |  |
| 3040 | G | GT | 2.8 | Insertion | *MT-RNR2* | rRNA |  |
| 3126 | CG | C | 3.9 | Deletion | *MT-RNR2* | rRNA |  |
| 3167 | TCC | T | 4.9 | Deletion | *MT-RNR2* | rRNA |  |
| 3167 | T | TC | 2.5 | Insertion | *MT-RNR2* | rRNA |  |
| 3244 | G | GC | 3.5 | Insertion | *MT-TL1* | tRNA |  |
| 3380 | GA | G | 5.4 | Deletion | *MT-ND1* | Coding | I |
| 3565 | A | AC | 2.1 | Insertion | *MT-ND1* | Coding | I |
| 3572 | T | TC | 5.8 | Insertion | *MT-ND1* | Coding | I |

**S11 Table. INDELs of MMNK-1 cell line (cont.)**

| Position | Ref | Variant | Heteroplasmy level (%) | INDEL | Maplocus | Category | OXPHOS  complex |
| --- | --- | --- | --- | --- | --- | --- | --- |
| 33 | CG | C | 10 | Deletion | MT-DLOOP2 | CR |  |
| 49 | AT | A | 2.9 | Deletion | MT-DLOOP2 | CR |  |
| 65 | T | TG | 4.6 | Insertion | MT-DLOOP2 | CR |  |
| 71 | GTA | G | 8.3 | Deletion | MT-DLOOP2 | CR |  |
| 145 | CT | C | 8.5 | Deletion | MT-DLOOP2 | CR |  |
| 302 | A | AC | 6.2 | Insertion | MT-DLOOP2 | CR |  |
| 310 | T | TC | 73.4 | Insertion | MT-DLOOP2 | CR |  |
| 432 | AC | A | 4.8 | Deletion | MT-DLOOP2 | CR |  |
| 455 | TC | T | 3.3 | Deletion | MT-DLOOP2 | CR |  |
| 460 | TC | T | 4.5 | Deletion | MT-DLOOP2 | CR |  |
| 493 | A | AC | 3.2 | Insertion | MT-DLOOP2 | CR |  |
| 499 | G | GC | 3.3 | Insertion | MT-DLOOP2 | CR |  |
| 513 | GCA | G | 99.2 | Deletion | MT-DLOOP2 | CR |  |
| 540 | A | AC | 3.9 | Insertion | MT-DLOOP2 | CR |  |
| 560 | CA | C | 12 | Deletion | MT-DLOOP2 | CR |  |
| 567 | A | AC | 3 | Insertion | MT-DLOOP2 | CR |  |
| 597 | C | CA | 2.8 | Insertion | *MT-TF* | tRNA |  |
| 704 | TC | T | 2 | Deletion | *MT-RNR1* | rRNA |  |
| 723 | AC | A | 3.5 | Deletion | *MT-RNR1* | rRNA |  |
| 749 | GA | G | 3.2 | Deletion | *MT-RNR1* | rRNA |  |
| 801 | A | AC | 3.7 | Insertion | *MT-RNR1* | rRNA |  |
| 808 | CG | C | 7.6 | Deletion | *MT-RNR1* | rRNA |  |
| 955 | AC | A | 5.2 | Deletion | *MT-RNR1* | rRNA |  |
| 961 | T | TC | 5.8 | Insertion | *MT-RNR1* | rRNA |  |
| 992 | T | TA | 4.2 | Insertion | *MT-RNR1* | rRNA |  |
| 1374 | A | AC | 5.5 | Insertion | *MT-RNR1* | rRNA |  |
| 1547 | TA | T | 4.8 | Deletion | *MT-RNR1* | rRNA |  |
| 1597 | CG | C | 8 | Deletion | *MT-RNR1* | rRNA |  |
| 1800 | GA | G | 3.8 | Deletion | *MT-RNR2* | rRNA |  |
| 1900 | AC | A | 20 | Deletion | *MT-RNR2* | rRNA |  |
| 1934 | T | TA | 2.2 | Insertion | *MT-RNR2* | rRNA |  |
| 2129 | GA | G | 3.8 | Deletion | *MT-RNR2* | rRNA |  |
| 2177 | T | TA | 5.4 | Insertion | *MT-RNR2* | rRNA |  |
| 2254 | CCT | C | 4.4 | Deletion | *MT-RNR2* | rRNA |  |
| 2347 | CA | C | 6 | Deletion | *MT-RNR2* | rRNA |  |
| 2456 | T | TA | 6.2 | Insertion | *MT-RNR2* | rRNA |  |
| 2465 | TA | T | 4.6 | Deletion | *MT-RNR2* | rRNA |  |
| 2487 | AC | A | 13.1 | Deletion | *MT-RNR2* | rRNA |  |
| 2496 | G | GT | 2.4 | Insertion | *MT-RNR2* | rRNA |  |
| 2813 | TG | T | 6.9 | Deletion | *MT-RNR2* | rRNA |  |
| 3027 | TA | T | 2.9 | Deletion | *MT-RNR2* | rRNA |  |
| 3040 | G | GT | 2.8 | Insertion | *MT-RNR2* | rRNA |  |
| 3126 | CG | C | 3.9 | Deletion | *MT-RNR2* | rRNA |  |
| 3167 | TCC | T | 4.9 | Deletion | *MT-RNR2* | rRNA |  |
| 3167 | T | TC | 2.5 | Insertion | *MT-RNR2* | rRNA |  |
| 3244 | G | GC | 3.5 | Insertion | *MT-TL1* | tRNA |  |
| 3380 | GA | G | 5.4 | Deletion | *MT-ND1* | Coding | I |
| 3565 | A | AC | 2.1 | Insertion | *MT-ND1* | Coding | I |
| 3572 | T | TC | 5.8 | Insertion | *MT-ND1* | Coding | I |

**S11 Table. INDELs of MMNK-1 cell line (cont.)**

| Position | Ref | Variant | Heteroplasmy level (%) | INDEL | Maplocus | Category | OXPHOS  complex |
| --- | --- | --- | --- | --- | --- | --- | --- |
| 3584 | AC | A | 5.6 | Deletion | *MT-ND1* | Coding | I |
| 3783 | CT | C | 7.4 | Deletion | *MT-ND1* | Coding | I |
| 4121 | GA | G | 3.4 | Deletion | *MT-ND1* | Coding | I |
| 4136 | AC | A | 15.4 | Deletion | *MT-ND1* | Coding | I |
| 4248 | TC | T | 3.7 | Deletion | *MT-ND1* | Coding | I |
| 4329 | C | CT | 2.2 | Insertion | *MT-TI* | tRNA |  |
| 4435 | AC | A | 4.4 | Deletion | *MT-TM* | tRNA |  |
| 4439 | CG | C | 2.9 | Deletion | *MT-TM* | tRNA |  |
| 4604 | C | CA | 2.2 | Insertion | *MT-ND2* | Coding | I |
| 4794 | GC | G | 5.8 | Deletion | *MT-ND2* | Coding | I |
| 4827 | CA | C | 4.9 | Deletion | *MT-ND2* | Coding | I |
| 4869 | CA | C | 5.8 | Deletion | *MT-ND2* | Coding | I |
| 4878 | GC | G | 6.2 | Deletion | *MT-ND2* | Coding | I |
| 4930 | T | TC | 2.2 | Insertion | *MT-ND2* | Coding | I |
| 5195 | CT | C | 4.9 | Deletion | *MT-ND2* | Coding | I |
| 5217 | T | TC | 5.7 | Insertion | *MT-ND2* | Coding | I |
| 5231 | GC | G | 12 | Deletion | *MT-ND2* | Coding | I |
| 5446 | T | TC | 2.3 | Insertion | *MT-ND2* | Coding | I |
| 5490 | CCT | C | 2.2 | Deletion | *MT-ND2* | Coding | I |
| 5507 | CT | C | 2.2 | Deletion | *MT-ND2* | Coding | I |
| 5763 | GC | G | 16.1 | Deletion | *MT-TC* | tRNA |  |
| 5829 | T | TA | 6.6 | Insertion | *MT-TY* | tRNA |  |
| 5851 | C | CT | 3.7 | Insertion | *MT-TY* | tRNA |  |
| 6152 | T | TC | 2.5 | Insertion | *MT-CO1* | Coding | IV |
| 6168 | GC | G | 2.7 | Deletion | *MT-CO1* | Coding | IV |
| 6185 | TC | T | 8.2 | Deletion | *MT-CO1* | Coding | IV |
| 6221 | T | TC | 2.8 | Insertion | *MT-CO1* | Coding | IV |
| 6223 | CCT | C | 2.2 | Deletion | *MT-CO1* | Coding | IV |
| 6446 | G | GC | 4.2 | Insertion | *MT-CO1* | Coding | IV |
| 6583 | A | AC | 6 | Insertion | *MT-CO1* | Coding | IV |
| 6611 | A | AT | 3.5 | Insertion | *MT-CO1* | Coding | IV |
| 6691 | G | GA | 6.9 | Insertion | *MT-CO1* | Coding | IV |
| 6942 | CT | C | 7.9 | Deletion | *MT-CO1* | Coding | IV |
| 7094 | T | TC | 4.7 | Insertion | *MT-CO1* | Coding | IV |
| 7180 | TC | T | 11 | Deletion | *MT-CO1* | Coding | IV |
| 7211 | GC | G | 5.4 | Deletion | *MT-CO1* | Coding | IV |
| 7341 | C | CG | 5.7 | Insertion | *MT-CO1* | Coding | IV |
| 7396 | G | GC | 2.1 | Insertion | *MT-CO1* | Coding | IV |
| 7446 | C | CA | 2.8 | Insertion | *MT-TS1* | tRNA |  |
| 7465 | A | AC | 3.1 | Insertion | *MT-TS1* | tRNA |  |
| 7514 | C | CA | 4.2 | Insertion | *MT-TS1* | tRNA |  |
| 7709 | C | CT | 2.8 | Insertion | *MT-CO2* | Coding | IV |
| 7954 | T | TC | 5.5 | Insertion | *MT-CO2* | Coding | IV |
| 8027 | GC | G | 5.4 | Deletion | *MT-CO2* | Coding | IV |
| 8035 | TC | T | 2.4 | Deletion | *MT-CO2* | Coding | IV |
| 8151 | CG | C | 11.1 | Deletion | *MT-CO2* | Coding | IV |
| 8232 | TA | T | 4.5 | Deletion | *MT-CO2* | Coding | IV |
| 8271 | AC | A | 3.8 | Deletion | NA | - |  |
| 8280 | AC | A | 4.7 | Deletion | NA | - |  |
| 8341 | AG | A | 3.7 | Deletion | *MT-TK* | tRNA |  |

**S11 Table. INDELs of MMNK-1 cell line (cont.)**

| Position | Ref | Variant | Heteroplasmy level (%) | INDEL | Maplocus | Category | OXPHOS  complex |
| --- | --- | --- | --- | --- | --- | --- | --- |
| 8354 | CT | C | 4.8 | Deletion | *MT-TK* | tRNA |  |
| 8368 | G | GC | 3.7 | Insertion | *MT-AYP8* | Coding | V |
| 8405 | A | AC | 3.1 | Insertion | *MT-AYP8* | Coding | V |
| 8490 | TA | T | 3.6 | Deletion | *MT-AYP8* | Coding | V |
| 8533 | G | GA | 3.4 | Insertion | *MT-ATP8* | Coding | V |
| 8557 | G | GC | 3.7 | Insertion | *MT-ATP8* | Coding | V |
| 8604 | TC | T | 8.8 | Deletion | *MT-ATP6* | Coding | V |
| 9449 | CG | C | 6.5 | Deletion | *MT-CO3* | Coding | IV |
| 9477 | G | GT | 5 | Insertion | *MT-CO3* | Coding | IV |
| 9531 | A | AC | 2.9 | Insertion | *MT-CO3* | Coding | IV |
| 9663 | GA | G | 5.9 | Deletion | *MT-CO3* | Coding | IV |
| 9721 | AT | A | 5.8 | Deletion | *MT-CO3* | Coding | IV |
| 9794 | AT | A | 7.6 | Deletion | *MT-CO3* | Coding | IV |
| 9994 | CT | C | 2.6 | Deletion | *MT-TG* | tRNA |  |
| 10047 | C | CA | 7.8 | Insertion | *MT-TG* | tRNA |  |
| 10089 | A | AC | 2.1 | Insertion | *MT-ND3* | Coding | I |
| 10191 | T | TC | 2.2 | Insertion | *MT-ND3* | Coding | I |
| 10204 | T | TC | 5.2 | Insertion | *MT-ND3* | Coding | I |
| 10272 | CT | C | 7.5 | Deletion | *MT-ND3* | Coding | I |
| 10357 | T | TA | 5.1 | Insertion | *MT-ND3* | Coding | I |
| 10380 | CA | C | 2.3 | Deletion | *MT-ND3* | Coding | I |
| 10879 | AT | A | 2.5 | Deletion | *MT-ND4* | Coding | I |
| 10916 | T | TC | 3 | Insertion | *MT-ND4* | Coding | I |
| 10935 | AC | A | 11.2 | Deletion | *MT-ND4* | Coding | I |
| 10946 | A | AC | 2.4 | Insertion | *MT-ND4* | Coding | I |
| 10978 | AC | A | 2 | Deletion | *MT-ND4* | Coding | I |
| 11031 | GAA | G | 5.3 | Deletion | *MT-ND4* | Coding | I |
| 11031 | G | GA | 2 | Insertion | *MT-ND4* | Coding | I |
| 11124 | TC | T | 2.4 | Deletion | *MT-ND4* | Coding | I |
| 11139 | T | TC | 2 | Insertion | *MT-ND4* | Coding | I |
| 11233 | T | TC | 3.7 | Insertion | *MT-ND4* | Coding | I |
| 11280 | TA | T | 5.4 | Deletion | *MT-ND4* | Coding | I |
| 11426 | GC | G | 2.7 | Deletion | *MT-ND4* | Coding | I |
| 11511 | AC | A | 2.7 | Deletion | *MT-ND4* | Coding | I |
| 11788 | CCT | C | 3.6 | Deletion | *MT-ND4* | Coding | I |
| 11826 | CT | C | 3.1 | Deletion | *MT-ND4* | Coding | I |
| 11866 | AC | A | 14.6 | Deletion | *MT-ND4* | Coding | I |
| 12005 | TG | T | 2.9 | Deletion | *MT-ND4* | Coding | I |
| 12083 | TC | T | 17.1 | Deletion | *MT-ND4* | Coding | I |
| 12135 | CT | C | 7.3 | Deletion | *MT-ND4* | Coding | I |
| 12232 | TC | T | 3 | Deletion | *MT-TS2* | tRNA |  |
| 12236 | GC | G | 3.1 | Deletion | *MT-TS2* | tRNA |  |
| 12305 | CA | C | 3.4 | Deletion | *MT-TL2* | tRNA |  |
| 12376 | TC | T | 4.4 | Deletion | *MT-ND5* | Coding | I |
| 12384 | T | TC | 2.6 | Insertion | *MT-ND5* | Coding | I |
| 12417 | C | CA | 8.2 | Insertion | *MT-ND5* | Coding | I |
| 12565 | TC | T | 3.2 | Deletion | *MT-ND5* | Coding | I |
| 12814 | GC | G | 4.2 | Deletion | *MT-ND5* | Coding | I |
| 13127 | AC | A | 12.2 | Deletion | *MT-ND5* | Coding | I |
| 13230 | C | CA | 3.8 | Insertion | *MT-ND5* | Coding | I |

**S11 Table. INDELs of MMNK-1 cell line (cont.)**

| Position | Ref | Variant | Heteroplasmy level (%) | INDEL | Maplocus | Category | OXPHOS  complex |
| --- | --- | --- | --- | --- | --- | --- | --- |
| 13646 | T | TC | 5 | Insertion | *MT-ND5* | Coding | I |
| 13753 | T | TC | 5.2 | Insertion | *MT-ND5* | Coding | I |
| 13781 | TC | T | 2.9 | Deletion | *MT-ND5* | Coding | I |
| 13820 | T | TC | 7.4 | Insertion | *MT-ND5* | Coding | I |
| 13980 | GC | G | 6.5 | Deletion | *MT-ND5* | Coding | I |
| 14074 | CA | C | 4.7 | Deletion | *MT-ND5* | Coding | I |
| 14109 | CT | C | 6.2 | Deletion | *MT-ND5* | Coding | I |
| 14154 | TC | T | 17.9 | Deletion | *MT-ND6* | Coding | I |
| 14157 | CCCG | C | 2.2 | Deletion | *MT-ND6* | Coding | I |
| 14264 | TC | T | 5.1 | Deletion | *MT-ND6* | Coding | I |
| 14339 | AC | A | 4.2 | Deletion | *MT-ND6* | Coding | I |
| 14395 | T | TA | 2.6 | Insertion | *MT-ND6* | Coding | I |
| 14488 | TC | T | 3 | Deletion | *MT-ND6* | Coding | I |
| 14503 | TA | T | 3.5 | Deletion | *MT-ND6* | Coding | I |
| 14530 | T | TC | 4.3 | Insertion | *MT-ND6* | Coding | I |
| 14611 | AG | A | 4.9 | Deletion | *MT-ND6* | Coding | I |
| 14754 | CA | C | 2.1 | Deletion | *MT-CYB* | Coding | III |
| 14808 | T | TC | 2.5 | Insertion | *MT-CYB* | Coding | III |
| 14813 | AC | A | 5.8 | Deletion | *MT-CYB* | Coding | III |
| 15224 | C | CT | 3.4 | Insertion | *MT-CYB* | Coding | III |
| 15254 | GT | G | 2.9 | Deletion | *MT-CYB* | Coding | III |
| 15262 | T | TC | 3.5 | Insertion | *MT-CYB* | Coding | III |
| 15383 | T | TC | 2 | Insertion | *MT-CYB* | Coding | III |
| 15448 | CT | C | 2 | Deletion | *MT-CYB* | Coding | III |
| 15536 | A | AC | 3.9 | Insertion | *MT-CYB* | Coding | III |
| 15541 | T | TC | 4 | Insertion | *MT-CYB* | Coding | III |
| 15961 | G | GA | 6.6 | Insertion | *MT-TP* | tRNA |  |
| 16189 | T | TC | 4.9 | Insertion | MT-DLOOP1 | CR |  |
| 16374 | AC | A | 3.5 | Deletion | MT-DLOOP1 | CR |  |
| 16431 | CA | C | 8 | Deletion | MT-DLOOP1 | CR |  |
| 16469 | TG | T | 4.3 | Deletion | MT-DLOOP1 | CR |  |

**S12 Table. Number of mtDNA structural variants of 10x, 30x, 50x and 100x cutoff.**

- **10X**

| Cell | KKU-023 | KKU-055 | KKU-100 | KKU-213A | KKU-452 | MMNK-1 |
| --- | --- | --- | --- | --- | --- | --- |
| DEL | 87 | 80 | 90 | 73 | 87 | 94 |
| DUP | 92 | 91 | 94 | 74 | 87 | 73 |
| INV | 97 | 107 | 105 | 73 | 100 | 115 |
| Total | 276 | 278 | 289 | 220 | 274 | 282 |

- **30X**

| Cell | KKU-023 | KKU-055 | KKU-100 | KKU-213A | KKU-452 | MMNK-1 |
| --- | --- | --- | --- | --- | --- | --- |
| DEL | 80 | 68 | 82 | 47 | 73 | 85 |
| DUP | 81 | 76 | 85 | 43 | 71 | 69 |
| INV | 82 | 87 | 90 | 46 | 80 | 95 |
| Total | 243 | 231 | 257 | 136 | 224 | 249 |

- **50X**

| - Cell | KKU-023 | KKU-055 | KKU-100 | KKU-213A | KKU-452 | MMNK-1 |
| --- | --- | --- | --- | --- | --- | --- |
| DEL | 66 | 59 | 72 | 25 | 57 | 74 |
| DUP | 69 | 55 | 74 | 25 | 46 | 58 |
| INV | 73 | 71 | 85 | 19 | 69 | 85 |
| Total | 208 | 185 | 231 | 69 | 172 | 217 |

- **100X**

| Cell | KKU-023 | KKU-055 | KKU-100 | KKU-213A | KKU-452 | MMNK-1 |
| --- | --- | --- | --- | --- | --- | --- |
| DEL | 47 | 28 | 49 | 5 | 23 | 44 |
| DUP | 46 | 30 | 50 | 8 | 23 | 40 |
| INV | 48 | 21 | 56 | 2 | 24 | 61 |
| Total | 141 | 79 | 155 | 15 | 70 | 145 |

**S13 Table. Depth coverages from BAM QC results.**

| Cell | Mean read quality (Nanoplot) | % mapped (qualimap) | Mean DP |
| --- | --- | --- | --- |
| KKU-023 | 12.4 | 85.83 | 9,320.87 |
| KKU-055 | 12.5 | 64.42 | 6,171.09 |
| KKU-100 | 12.3 | 98.23 | 9,134.75 |
| KKU-213A | 12.7 | 34.76 | 2,706.66 |
| KKU-452 | 12.2 | 68.16 | 5,869.44 |
| MMNK-1 | 12.4 | 84.13 | 8,845.85 |

**S14 Table. Haplogroup, contamination level, quality, and QC results from mitoverse.**

| Cell line | Mean coverage | Contamination level (%) | Quality | QC | Major Haplogroup |
| --- | --- | --- | --- | --- | --- |
| KKU-023 | 2003.26 | 0.025 | 0.9559 | Pass | R9b1a2b*2 |
| KKU-055 | 2001.22 | 0.034 | 0.9577 | Pass | F1a1a1 |
| KKU-100 | 1998.79 | 0.023 | 0.9713 | Pass | M74b2 |
| KKU-213A | 1996.45 | 0.054 | 0.9503 | Pass | C7a |
| KKU-452 | 1990.96 | 0.027 | 0.7976 | Pass | M12b |
| MMNK-1 | 1997.1 | 0.025 | 0.9912 | Pass | M7c1a2a1 |

**S15 Table. Doubling time, total number of SNV and INDEL of each cell line.**

| **Cell line** | **Doubling time (h)** | **Total SNVs** | **Total INDELs** |
| --- | --- | --- | --- |
| KKU-023 | 34.8 | 48 | 181 |
| KKU-055 | 24 | 59 | 196 |
| KKU-100 | 72 | 61 | 174 |
| KKU-213A | 23 | 91 | 198 |
| KKU-452 | 17.9 | 69 | 160 |
| MMNK-1 | 40 | 45 | 180 |

**S16 Table. Droplet digital PCR results of IL-6, CK-19, and β-actin concentration (cp/µL).**

| **Sample** | **IL-6 concentration (cp/µL)** | **CK19 concentration (cp/µL)** | **β-actin concentration (cp/µL)** |
| --- | --- | --- | --- |
| KKU-023 | 33.35 | 30.52 | 6666.7 |
| KKU-055 | 11.82 | 3.83 | 6486.3 |
| KKU-100 | 125.7 | 7.722 | 25500 |
| KKU-213A | 3785.5 | 72.38 | 24500 |
| KKU-452 | 77.72 | 0.426 | 53100 |
| MMNK-1 | 4582.6 | 84.21 | 73000 |


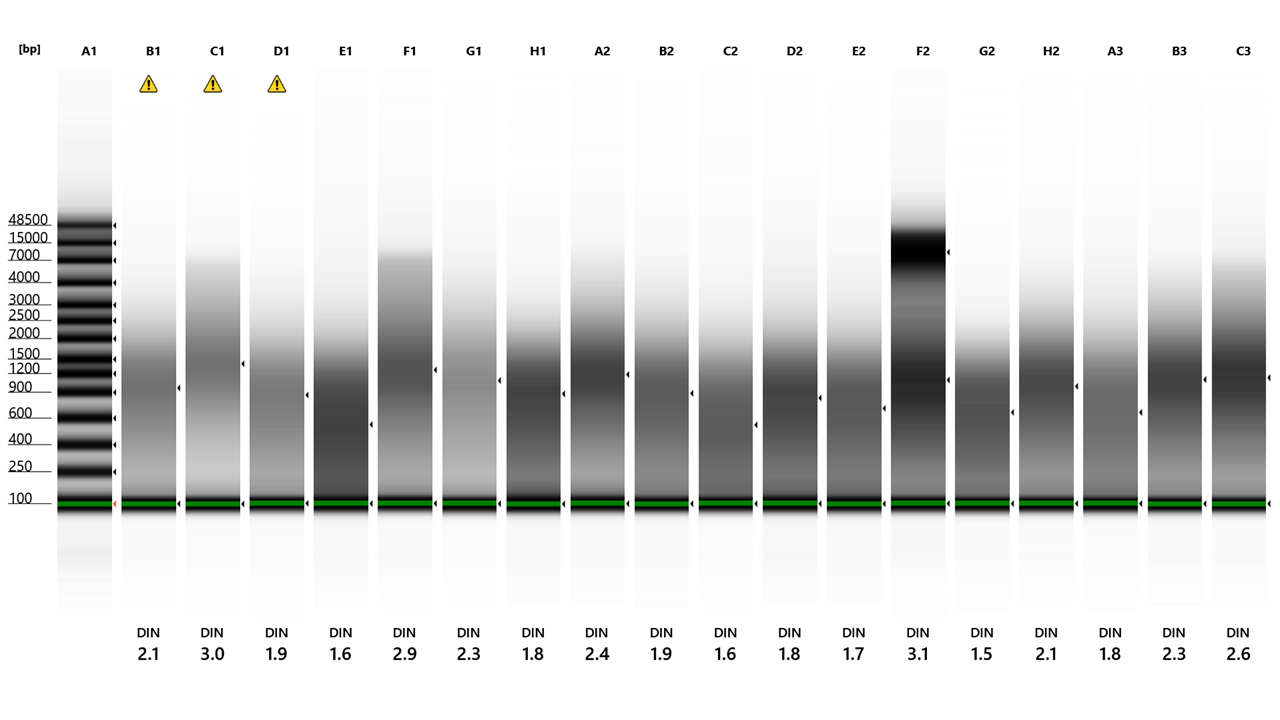


**S1 Fig. The gel interface on TapeStation Analysis of each mtDNA sample.** The following sample labels were assigned to each corresponding position: A1: ladder; B1: KKU-213A-1; C1: KKU-213A-2; D1: KKU-213A-3; E1: KKU-100-1; F1: KKU-100-2; G1: KKU-100-3; H1: KKU-055-1; A2: KKU-055-2; B2: KKU-055-3; C2: KKU-452-1; D2: KKU-452-2; E2: KKU-452-3; F2: KKU-023-1; G2: KKU-023-2; H2: KKU-023-3; A3: MMNK1-1; B3: MMNK1-2; C3: MMNK1-3.


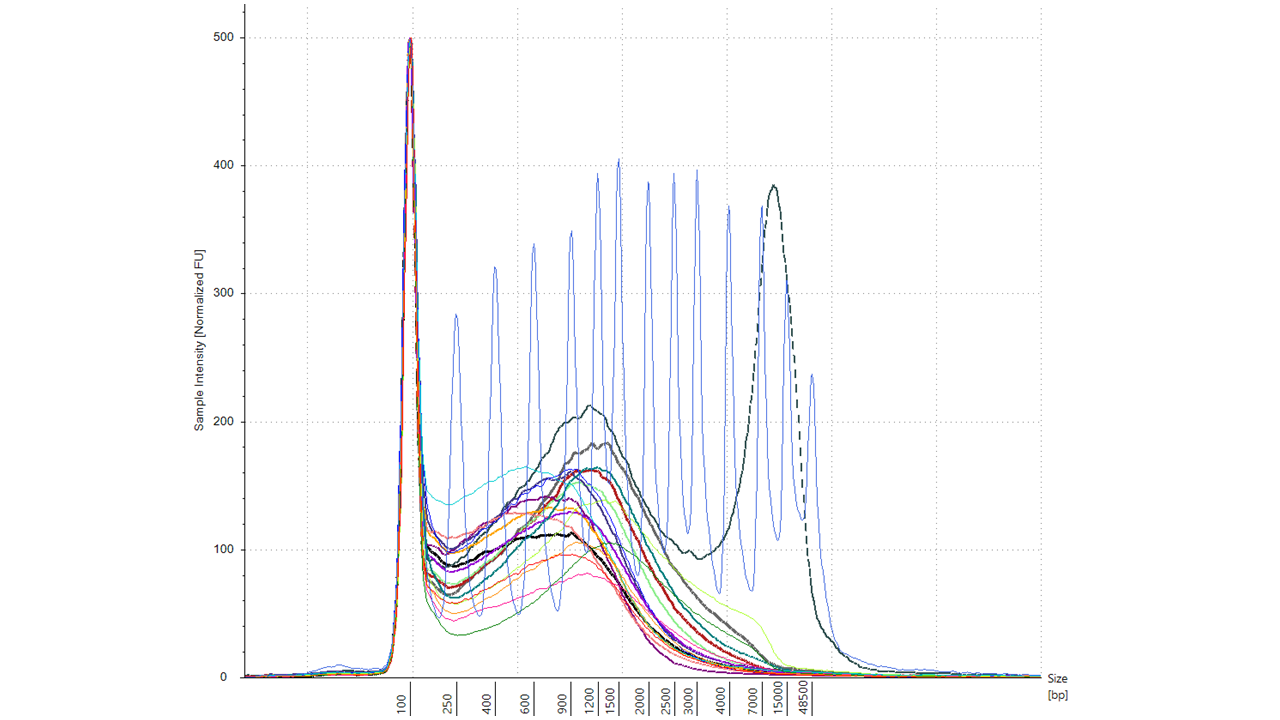


**S2 Fig. The electropherogram of each mtDNA sample.** It displays the sample peaks (range 319 - 3010 pb) as well as the marker peaks (100 bp).


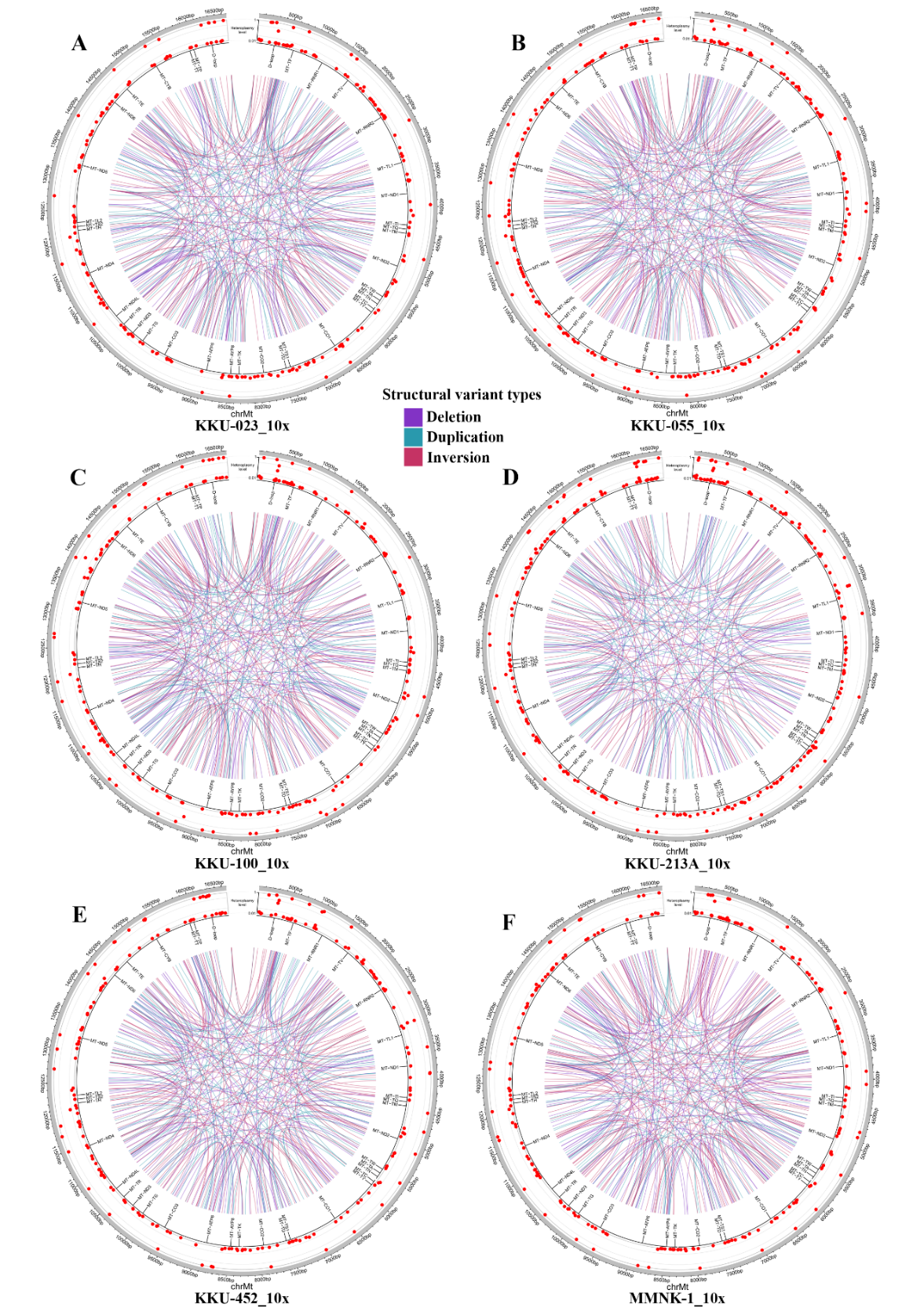


**S3 Fig. Circos plot of 10X depth coverage cutoff.** The circos plots depict the positions of SNVs and INDELs, marked by red dots along the circumference. Structural deletions are indicated by violet lines, structural duplications by blue-green lines, and structural inversions by red-wine lines.

**
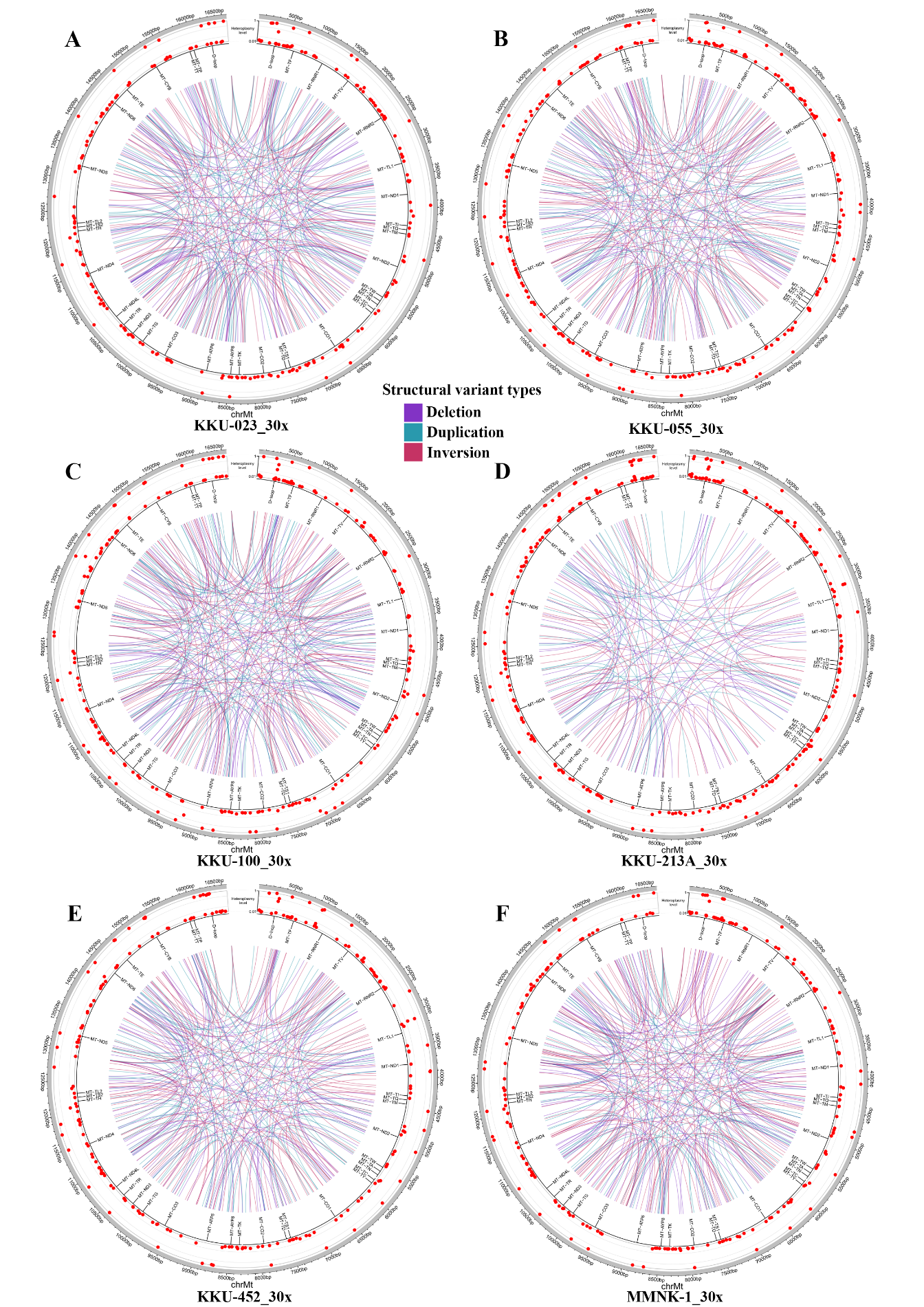
**

**S4 Fig. Circos plot of 30X depth coverage cutoff.**

**
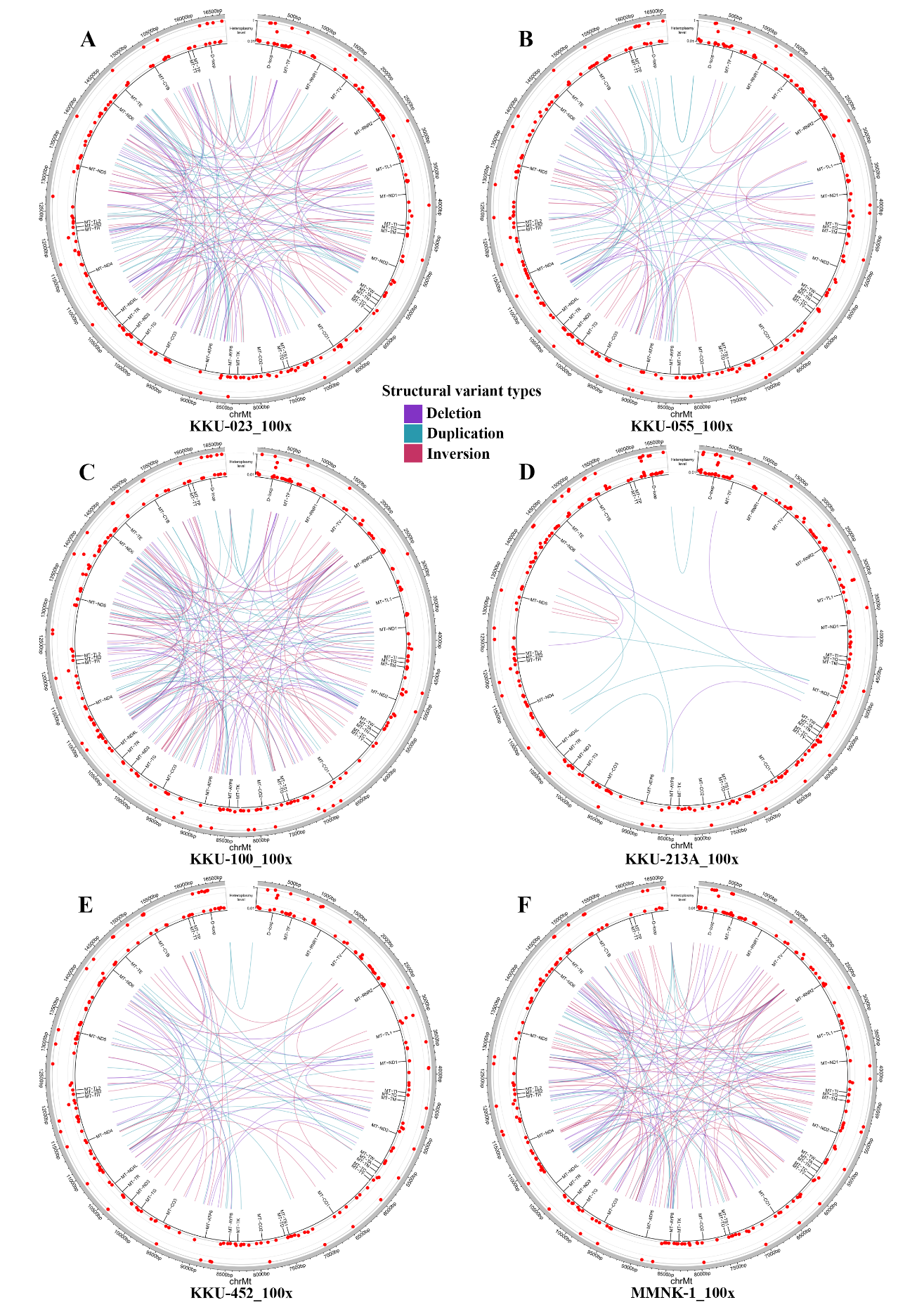
**

**S5 Fig. Circos plot of 100X depth coverage cutoff.**


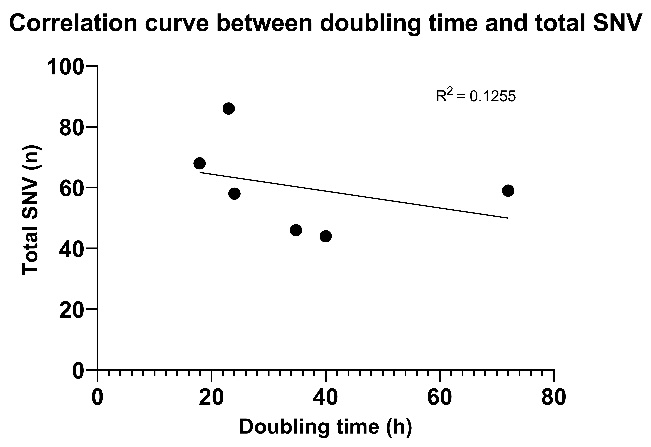

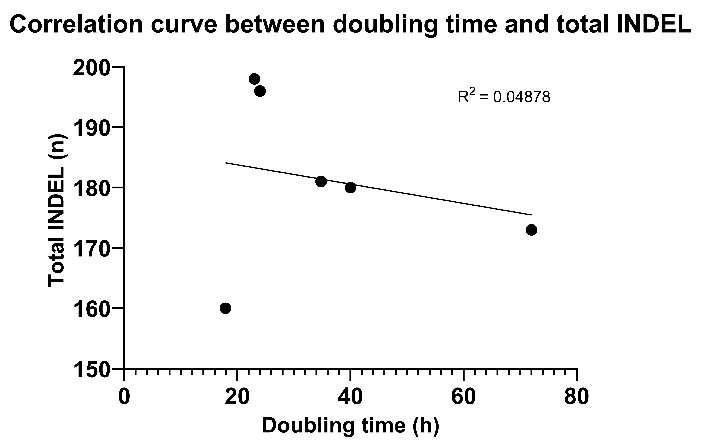


**S6 Fig. Correlation curve between doubling time and total number of SNV and INDEL.**

**
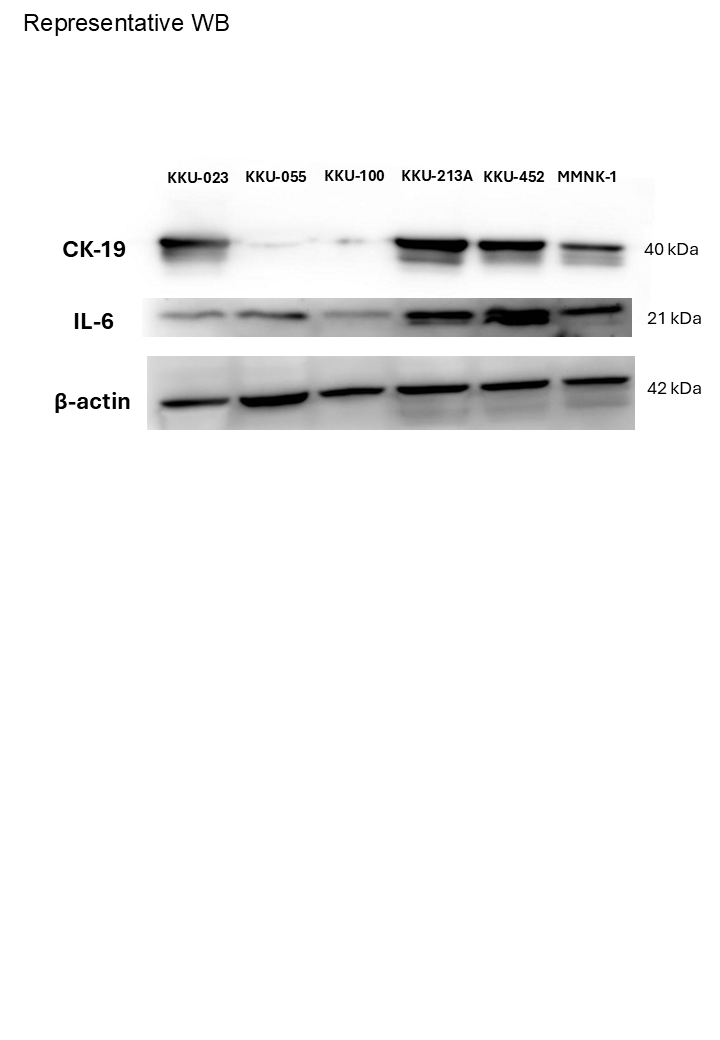
**

**S7 Fig.** **The representative of western blot analysis shows the expression of CK-19 and IL-6 in each cell line.**

**
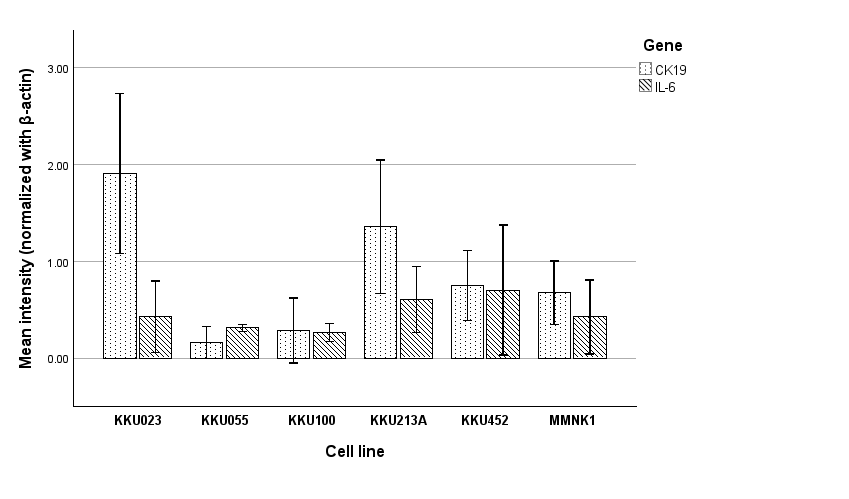
**

**S8 Fig. Western Blotting intensity graph of CK-19 and IL-6 normalized with β-actin.** Three independent experiments have been performed.

**
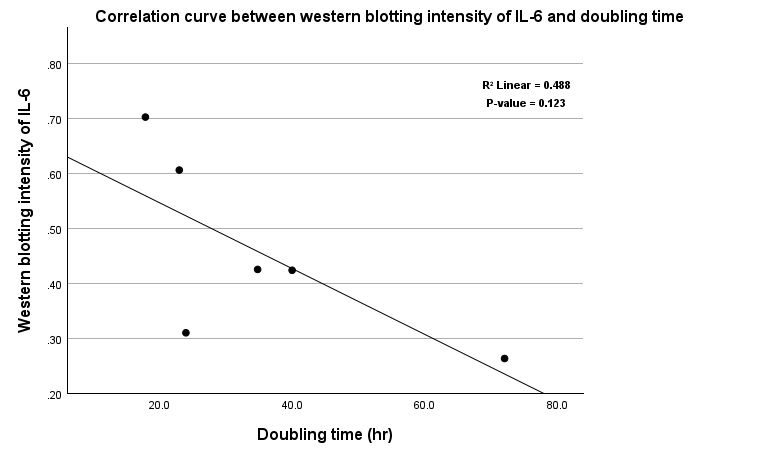
**

**
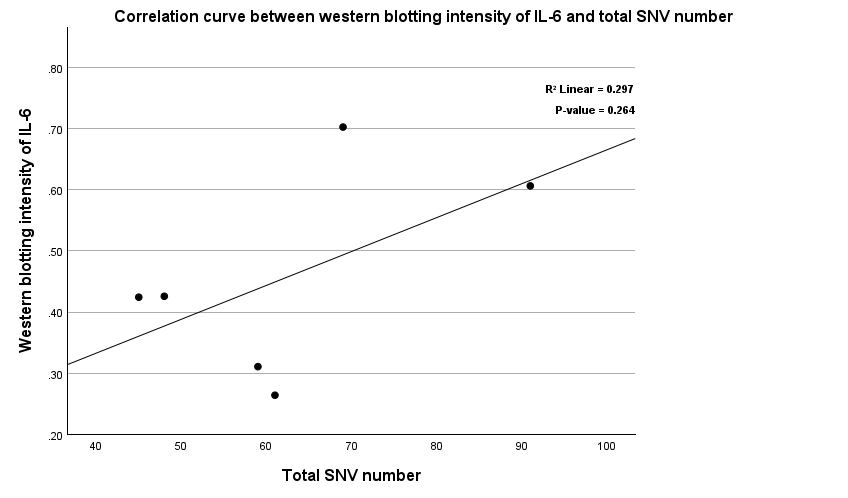
**

**
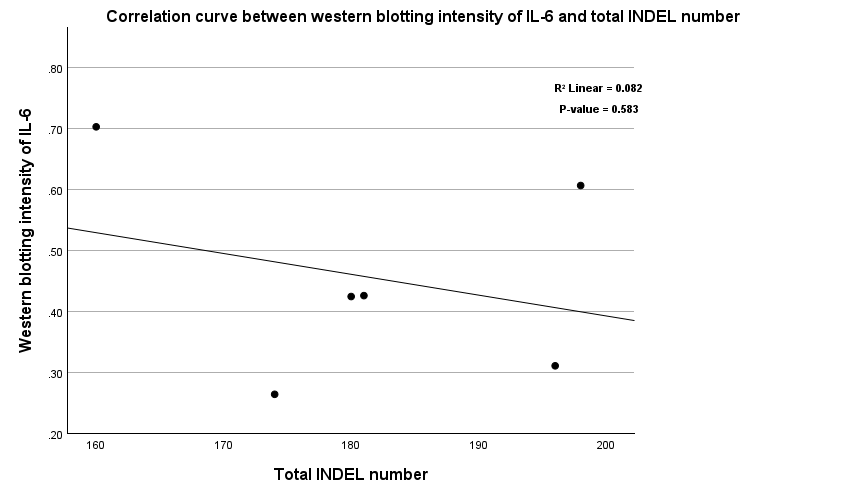
**

**S9 Fig. Correlation curves of IL-6 western blotting intensity with doubling time, SNVs, and INDELs.**

**
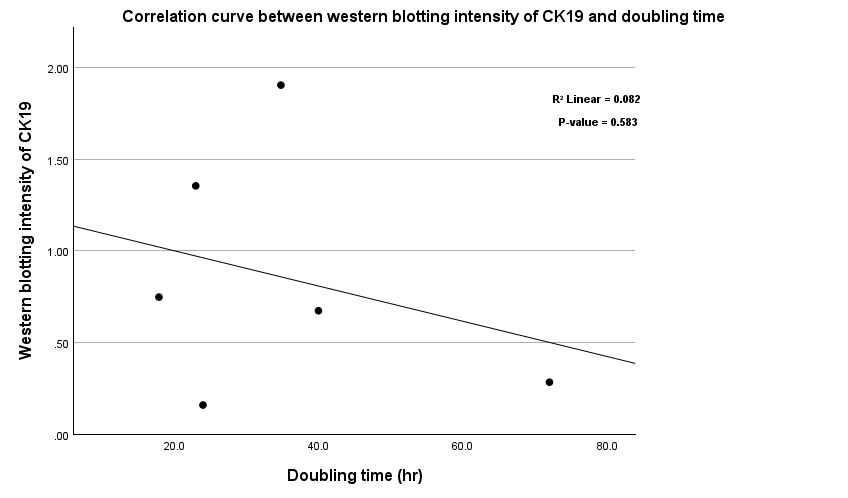
**

**
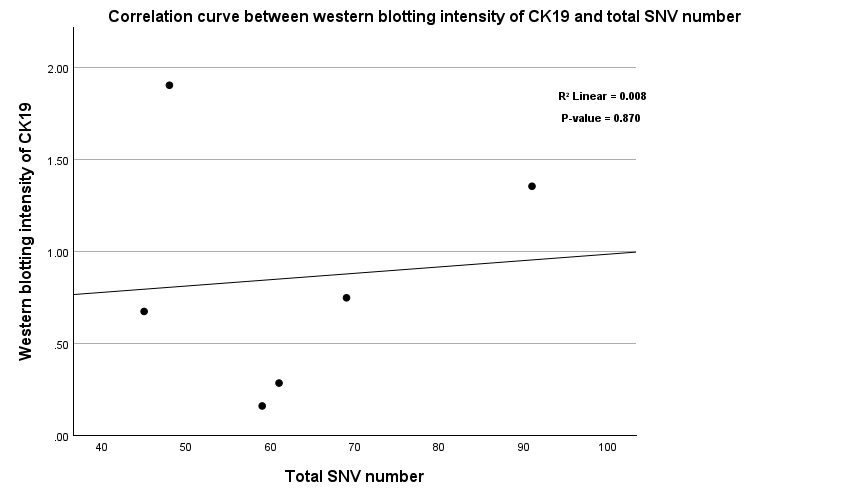
**

**
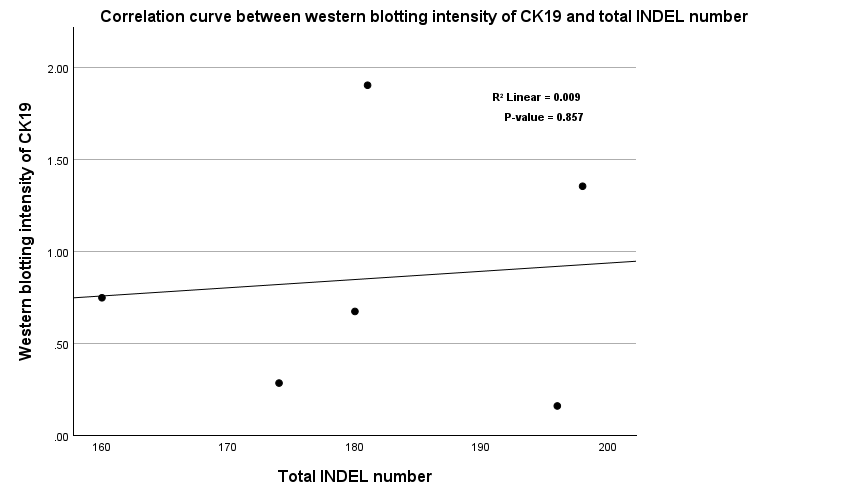
**

**S10 Fig. Correlation curves of CK-19 western blotting intensity with doubling time, SNVs, and INDELs.**


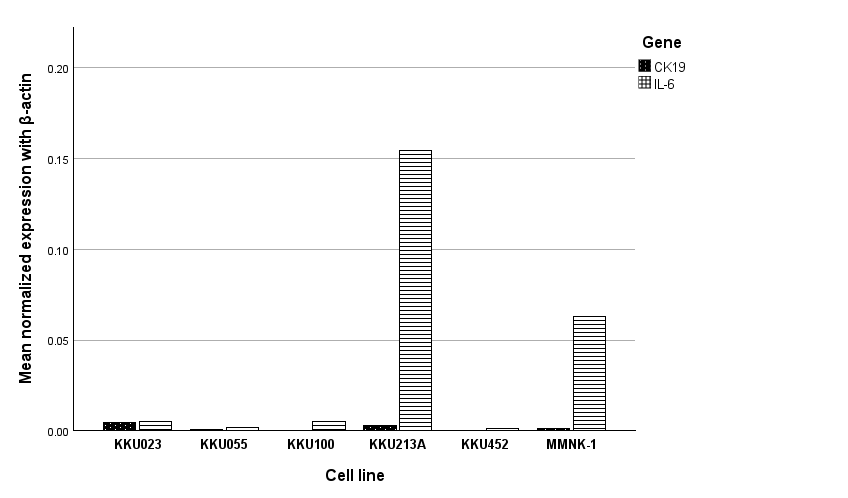


**S11 Fig. Gene expression level of CK-19 and IL-6 after normalized with β-actin of each cell line demonstrated by droplet digital PCR.**


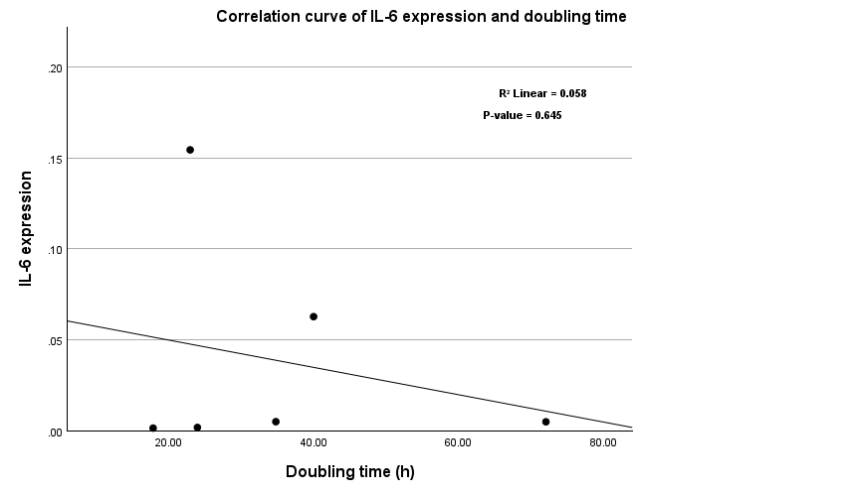


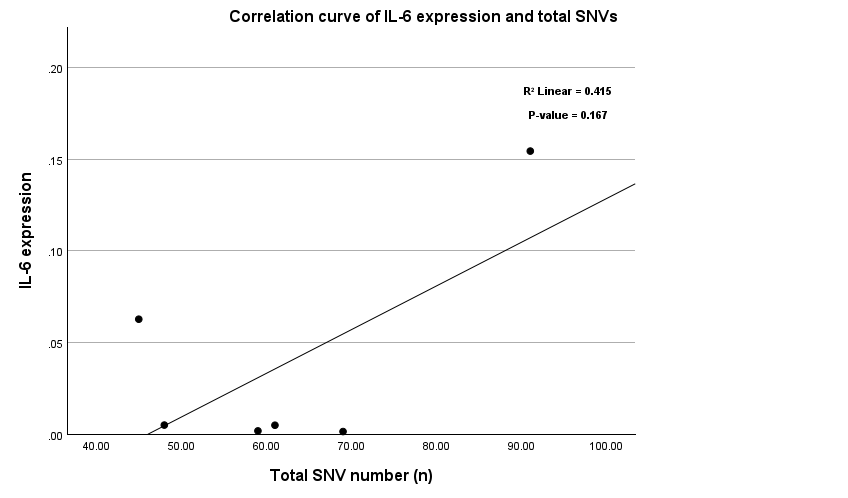


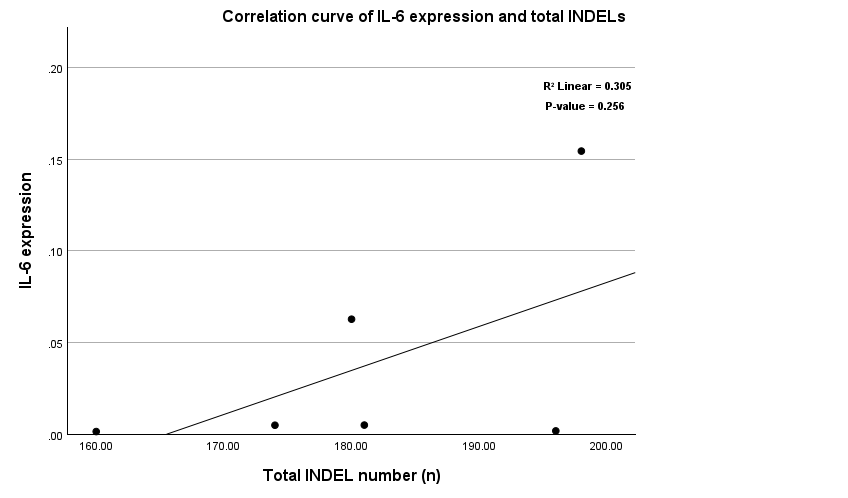


**S12 Fig. Correlation curves of IL-6 gene expression with doubling time, SNVs, and INDELs.**


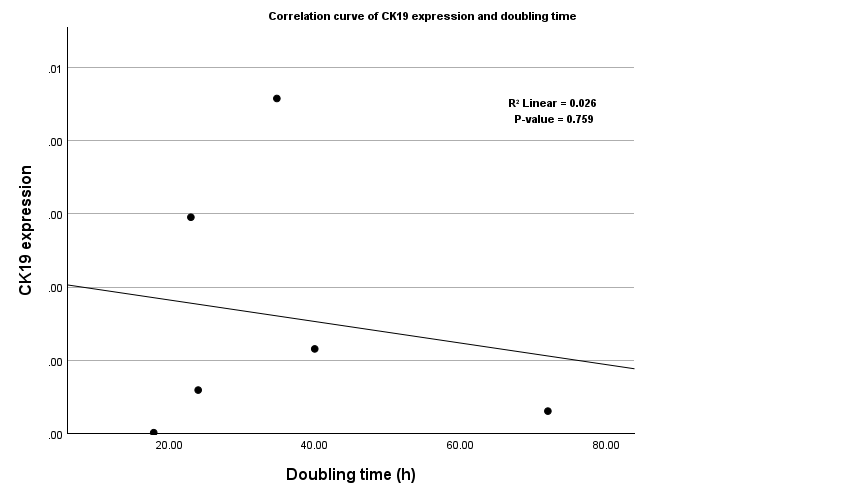
**
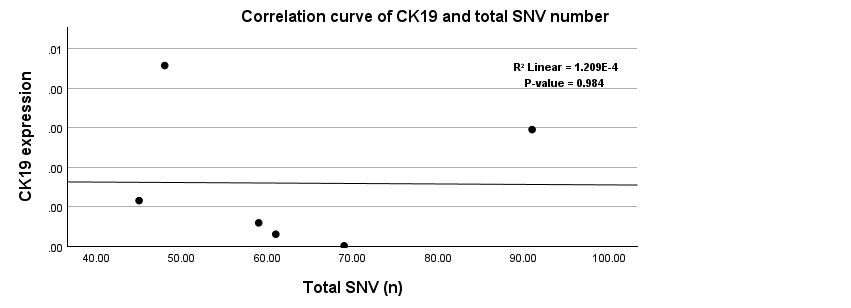
**

**
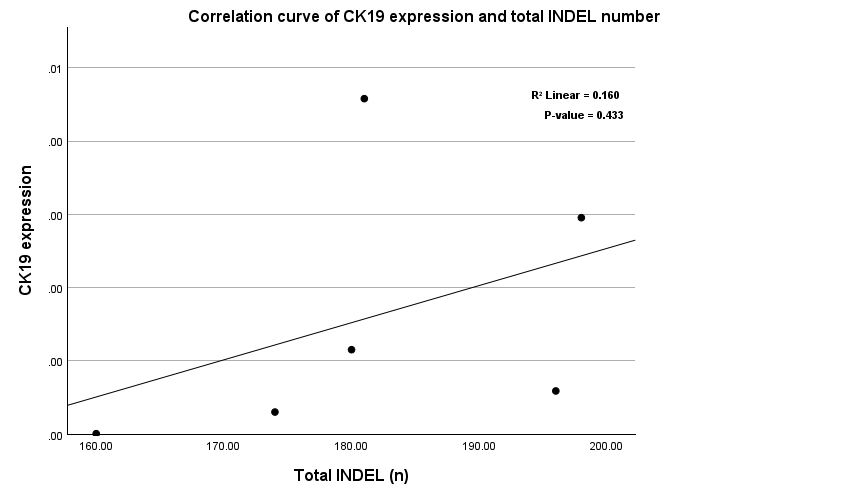
**

**S13 Fig. Correlation curves of CK19 gene expression with doubling time, SNVs, and INDELs.**
