# Supplementary material for: Mitochondrial genomic alterations in cholangiocarcinoma cell lines
Source: PLoS One. 2025 Jun 9;20(6):e0323844. doi: 10.1371/journal.pone.0323844 (PMC12148147; doi:10.1371/journal.pone.0323844)
Supplement: Attachment — Submitted filename: PONE-D-24-41324R1 Response to reviewers.docx [file pone.0323844.s003.docx]

22^nd^ November, 2024

RE: PONE-D-24-41324

Dear Editor-in-Chief,

We are very thankful again to you and the reviewers for the critical reading of our manuscript entitled “**Mitochondrial genomic alterations in cholangiocarcinoma cell lines**”, by Faipan et al., to PLOS ONE. We appreciate the reviewers’ comments and suggestions, and have incorporated them into the manuscript as follows:

**1. To establish the functional significance of the observed mtDNA mutations, the authors should evaluate the expression levels of the genes harboring these mutations through qPCR and confirm protein expression via western blotting. These analyses would provide crucial information on whether the identified mutations result in corresponding changes at the transcriptional or translational levels.**

**Ans**: Thank you for your suggestion. There is the previous study demonstrated that the KKU-213A CCA cell line, which harbors numerous mtDNA SNVs, had ALDH1A3 expression which significantly rises under lactic acidosis (LA) conditions, and correlates with LDHA expression. Higher ALDH1A3 levels have been linked to poorer patient survival, indicating its potential as a prognostic marker, which we have added into the discussion part line 405 to 409 as highlighted in blue (Thamrongwaranggoon et al., 2022). We also assessed the expression of key proteins, including CK19 and IL-6, using Western blotting to investigate potential functional consequences of the observed mtDNA mutations. Our results show that KKU-023, KKU-213A, and KKU-452 exhibit high expression levels of both CK19 and IL-6 proteins, while KKU-055, KKU-100, and MMNK-1 demonstrate lower expression levels (See Response to Reviewer No. 3 below). Moreover, to explore the potential link between mtDNA mutations and protein expression, we analyzed the correlation between CK19 and IL-6 protein expression level and the total number of mtDNA single nucleotide variants (SNVs) and insertions-deletions (INDELs). No significant correlations were found between mtDNA mutations and CK19 protein expression levels, aligning with our literature review, which revealed no prior studies addressing this relationship. In contrast, IL-6 protein expression has been linked to TFB2M, a key regulator of mitochondrial DNA (mtDNA) transcription and maintenance. Overexpression of TFB2M has been shown to increase extracellular mtDNA and IL-6 levels in ovarian cancer cells, promoting M2 macrophage infiltration via the cytoplasmic mtDNA/TLR9/NF-κB/IL-6 pathway (Lei et al., 2024). Although our results did not demonstrate a significant association. We have added this information in the discussion part line 409 to 413 highlighted in blue.

**Reference**

Lei, T., Rui, Y., Xiaoshuang, Z., Jinglan, Z., & Jihong, Z. (2024). Mitochondria transcription and cancer. *Cell Death Discovery*, *10*(1), 168. https://doi.org/10.1038/s41420-024-01926-3

Thamrongwaranggoon, U., Detarya, M., Seubwai, W., Saengboonmee, C., Hino, S., Koga, T., Nakao, M., & Wongkham, S. (2022). Lactic acidosis promotes aggressive features of cholangiocarcinoma cells via upregulating ALDH1A3 expression through EGFR axis. *Life Sciences*, *302*, 120648. https://doi.org/10.1016/j.lfs.2022.120648

**2. The manuscript would benefit from the inclusion of phenotypic characterization of the CCA-derived cell lines. Specifically, conducting colony formation assays and determining the doubling time of each cell line would offer important insights into their proliferative and clonogenic capacities, further contributing to understanding their oncogenic potential.**

**Ans**: Thank you for your insightful suggestion. The doubling time and the total number of mtDNA SNVs and INDELs for each cell line have already been provided in the table below. We also analyzed the correlation between them, unfortunately no significant correlation was observed. We put this sentence in the discussion part as in line 415-417 as highlighted in blue.

| **Cell line** | **Doubling time (h)** | **Total number of SNVs** | **Total number of INDELs** |
| --- | --- | --- | --- |
| KKU-023 | 34.8 | 46 | 181 |
| KKU-055 | 24 | 58 | 196 |
| KKU-100 | 72 | 59 | 173 |
| KKU-213A | 23 | 86 | 198 |
| KKU-452 | 17.9 | 68 | 160 |
| MMNK-1 | 40 | 44 | 180 |

P value of correlation curve between doubling time and total INDEL and total SNV is 0.6741 and 0.4909, respectively.

**3. It is also recommended to assess the expression of cholangiocarcinoma-related genes by qPCR, including markers such as CK19, MUC1, MUC5, SOX9, EPCAM, IL6, TGFB1, CXCL10, CD133, UHRF1, and SOX17. This would provide a more comprehensive molecular characterization of the cell lines and allow for better contextualization within the CCA biology landscape.**

**Ans**: Thank you for your valuable suggestion. We assessed the expression of CK19 and IL-6 at the protein level using western blot analysis. Our results demonstrate that KKU-023, KKU-213A, and KKU-452 cell lines exhibited high CK19 and IL-6 protein expression, while KKU-055, KKU-100, and MMNK-1 showed low expression levels, as illustrated by the protein intensity bar graphs in Figure 3 below. Additionally, we constructed a correlation curve to evaluate the relationship between CK19 and IL-6 protein intensities and the total number of mtDNA single SNVs and INDELs (Figure 3. And 4.). However, this analysis did not reveal significant correlations.We acknowledge the importance of a more comprehensive molecular characterization and will consider expanding our analysis to include the suggested cholangiocarcinoma-related genes in future studies.

**Figure 1.** Western Blot analysis of CK19 and IL-6 proteins

**Figure 2.** CK19 and IL-6 protein intensity of each cell lines

**Figure 3.** Correlation between CK19 intensity and total mtDNA INDELs and SNVs

**Figure 4.** Correlation between IL-6 intensity and total mtDNA INDELs and SNVs

**4. If formalin-fixed, paraffin-embedded tissue samples from the original patient tumors are available (or from another cohort of patients as a validation cohort), performing immunohistochemistry to assess the expression of the corresponding proteins would be highly informative. This would facilitate the correlation of genomic alterations with potential protein-level dysregulation in the tumor tissues.**

**Ans**: Thank you so much for a great suggestion. We are now conducting the mitochondria mutation analysis in the lager number of cholangiocarcinoma tissues and your suggestion will be absolutely performed.

**5. Additionally, should clinical data from the patient donors be accessible, it would be beneficial to include a description of the phenotypic characteristics of each CCA donor sample, such as tumor grade, stage, and clinical outcomes. This would significantly enhance the clinical relevance of the findings.**

**Ans**: Thank you for the suggestion. We have added the table of the clinical characteristics of the patient donors from whom the cholangiocarcinoma (CCA) cell lines as in Table 8 of the revised version.

**6. Lastly, the authors could consider performing association analyses between the detected mutations and clinical features of cholangiocarcinoma using publicly available datasets, such as those from The Cancer Genome Atlas (TCGA). Using these data could provide valuable correlations between the mutations identified and key clinical parameters, such as patient survival rates, thereby strengthening the translational impact of the study.**

**Ans**: We respectfully note that TCGA primarily focuses on nDNA data, including somatic and germline mutations, gene expression, and other features related to nuclear genomics. While TCGA has been invaluable for understanding various aspects of cancer biology, it is not well-suited for the detailed investigation of mtDNA mutations. Unfortunately, the TCGA data does not provide direct or comprehensive coverage of mtDNA as its sequencing methods prioritize nuclear genomes. Although some mtDNA reads can be indirectly extracted from whole-genome or whole-exome sequencing data, these reads are often limited, lack sufficient depth, and can result in incomplete or biased mtDNA mutation profiles. Therefore, conducting meaningful association analyses between mtDNA mutations and clinical features using TCGA data would not be feasible or accurate for this study's specific focus on mitochondrial genomics in CCA. Based on this limitation, we have chosen to rely on direct sequencing and analysis of mtDNA from CCA cell lines in our investigation to ensure robust and accurate identification of mtDNA variants. We believe this approach is more appropriate for achieving the study's aims.

We have also added Hasaya Dokduang as the co-author in the revised manuscript because she is the person who handles western blot analysis, which is shown in this response to reviewers’ letter.

We believe that the manuscript has been improved satisfactorily and hope that it is now acceptable for publication in PLOS ONE*.*

Yours Sincerely,

Watcharin Loilome, Ph.D.

Department of Systems Biosciences and Computational Medicine

Faculty of Medicine,

Khon Kaen University, Khon Kaen, 40002, THAILAND

Email: [watclo@kku.ac.th](mailto:watclo@kku.ac.th)
