# Supplementary material for: Mitochondrial genomic alterations in cholangiocarcinoma cell lines
Source: PLoS One. 2025 Jun 9;20(6):e0323844. doi: 10.1371/journal.pone.0323844 (PMC12148147; doi:10.1371/journal.pone.0323844)
Supplement: Attachment — Submitted filename: PONE-D-24-41324R2 Response to reviewers.docx [file pone.0323844.s004.docx]

**Response to reviewers**

1. **Western Blot Data as Supplementary Material: The Western blot results should be included as supplementary material. Additionally, if not already done, Western blot intensity quantifications should be normalized to β-actin to ensure accurate comparisons. The supplementary material should include the corrected quantification graphs and the corresponding Western blot images. I think it is interesting to have this first molecular characterization. Even if nonsignificant results were found, there are differences in the expression of CK19 specially. The manuscript should mention that further molecular characterization is needed. How many replicates were performed? data are presented as bars with SD or SEM and is impossible to infer the number of replicates.**

**Author’s response**: Thank you for your valuable feedback. We have already performed three independent western blot analysis and intensity normalization to β-actin and included the quantification graphs (Figure S8) along with the corresponding representative western blot images (Figures S7) in the supplementary material. The correlation between IL-6 and CK-19 western blotting intensity with doubling time, SNVs, and INDELs are also provided (Figure S9-10) We also provide the raw data of the three independent western blot analysis as below.

|  |  |  |
| --- | --- | --- |

2. **qPCR Expression Analysis and Correlations: The expression levels of the suggested markers should be evaluated by qPCR. These data should then be used for correlation analyses with doubling time, SNVs, and INDELs. Since qPCR is a straightforward measurement, these additional data would provide valuable support for the characterization, making the findings more robust rather than purely observational.**

**Author’s response**: Thank you for your valuable suggestion. We appreciate your recommendation to include quantitative expression analysis to strengthen our findings.

In response, we have performed droplet digital PCR (ddPCR) to quantitatively assess the expression levels of IL-6, CK19, and β-actin across our cell lines. These data are presented in Table S16. Gene expression levels of IL-6 and CK19 were normalized to β-actin for each cell line (Figure S11). Subsequently, we conducted correlation analyses between the normalized gene expression and key cellular features, including doubling time, SNVs, and INDELs. The corresponding correlation plots are shown in Figures S12 and S13. Although the correlation analyses did not yield statistically significant results, likely due to the limited number of cell lines available, we believe the inclusion of these data adds quantitative depth to our study and provides a basis for future investigations with larger sample sizes.

**S16 Table. Droplet digital PCR results of IL-6, CK-19, and β-actin concentration**

| **Sample** | **IL-6 concentration (cp/µL)** | **CK19 concentration (cp/µL)** | **β-actin concentration (cp/µL)** |
| --- | --- | --- | --- |
| KKU-023 | 33.35 | 30.52 | 6666.7 |
| KKU-055 | 11.82 | 3.83 | 6486.3 |
| KKU-100 | 125.7 | 7.722 | 25500 |
| KKU-213A | 3785.5 | 72.38 | 24500 |
| KKU-452 | 77.72 | 0.426 | 53100 |
| MMNK-1 | 4582.6 | 84.21 | 73000 |

**S11 Fig. Gene expression level of CK-19 and IL-6 after normalized with β-actin of each cell line demonstrated by droplet digital PCR.**

**S12 Fig. Correlation curves of IL-6 gene expression with doubling time, SNVs, and INDELs, respectively.**

**S13 Fig. Correlation curves of CK19 gene expression with doubling time, SNVs, and INDELs, respectively.**
